# Carrier‐Free Berberine/Nitidine Chloride Self‐Assembled Nanoparticles Induce Ferroptosis to Overcome Bortezomib Resistance in Multiple Myeloma

**DOI:** 10.1002/advs.77174

**Published:** 2026-08-18

**Authors:** Yiwen Lv, Shuang Liu, Hong Wu, Guiluan Li, Ying Rong, Guangchao Li, Yangmin Zhu, Qi Zhong, Ruiming Ou, Huijuan Shen, Zhi Liu, Jing Huang, Zhenwei Wang, Pei Lin, Qing Zhang, Guopan Yu, Zhao Yin

**Affiliations:** ^1^ The First Affiliated Hospital of Guangzhou University of Chinese Medicine Guangzhou People's Republic of China; ^2^ The Affiliated Guangdong Second Provincial General Hospital of Jinan University Guangzhou Guangdong People's Republic of China; ^3^ Key Laboratory of Regenerative Medicine of Ministry of Education Guangzhou Guangdong People's Republic of China; ^4^ Guangdong Province Key Laboratory of Pharmacodynamic Constituents of TCM and New Drugs Research College of Pharmacy Jinan University Guangzhou People's Republic of China

**Keywords:** bortezomib resistance, carrier‐free self‐assembly, ferroptosis, multiple myeloma, natural products

## Abstract

Therapeutic relapse driven by bortezomib resistance represents a formidable clinical barrier in the management of multiple myeloma (MM). Here, a carrier‐free, supramolecular nanoplatform was engineered through the spontaneous co‐assembly of two natural alkaloids, berberine (BBR) and nitidine chloride (NC), termed BBR/NC‐SAPs, to counter this malignancy. Driven by cooperative π‐π stacking and van der Waals forces, BBR/NC‐SAPs display superior anti‐MM efficacy and optimized biosafety as compared to the free‐drug combination. Quantitative proteomics and functional landscapes showed that BBR/NC‐SAPs robustly activate iron‐dependent ferroptosis, as evidenced by massive lipid peroxidation, intracellular Fe^2+^ overload, and mitochondrial depolarization. Mechanistically, integrated target deconvolution and atomistic molecular modeling identified NR2F2 as a direct target of BBR/NC‐SAPs. Functional knock‐out and rescue evaluations definitively validated that NR2F2 is an essential mediator governing the therapeutic response, as NR2F2 depletion occluded the ferroptotic cascade, and lentiviral reconstitution fully restored cellular sensitivity and GPX4‐associated ferroptosis activation. Further, BBR/NC‐SAPs markedly suppressed tumor growth and prolonged survival in bortezomib‐resistant MM xenograft models, a therapeutic benefit actively reversed by the ferroptosis inhibitor ferrostatin‐1. Collectively, this work highlights a paradigm of natural product‐derived nanotechnology that targets the NR2F2–GPX4 axis, offering a promising supramolecular strategy to overcome drug resistance in refractory hematological tumors.

## Graphical abstract

A schematic illustrating the fabrication process and ferroptosis‐mediated therapeutic mechanism of carrier‐free self‐assembled BBR/NC‐SAPs for overcoming bortezomib resistance in multiple myeloma.

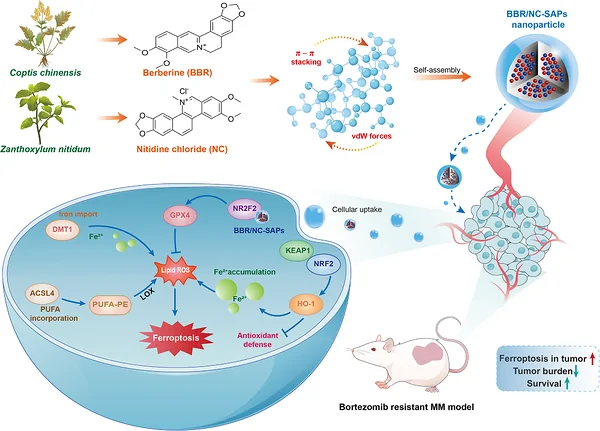

**Abbreviations**
3‐MA: 3‐Methyladenine
NAC: Acetylcysteine
BBR: Berberine
BP: Biological Process
BLI: Bioluminescence imaging
CC: Cellular Component
CETSA: Cellular Thermal Shift Assay
DLS: Dynamic light scattering
ECL: Enhanced chemiluminescence
FTIR: Fourier‐transform infrared
LBD: Ligand‐binding domain
Lip‐ROS: Lipid reactive oxygen species
MFI: Mean fluorescence intensity
MD: Molecular dynamics
MF: Molecular Function
MM: Multiple myeloma
NC: Nitidine chloride
NR2F2: Nuclear receptor subfamily 2 group F member 2
PBS: Phosphate‐buffered saline
PDI: Polydispersity index
SAPs: Self‐assembled nanoparticles
sgRNAs: Single‐guide RNAs
SPR: Surface Plasmon Resonance
SPR–HPLC–MS: Surface plasmon resonance coupled with high‐performance liquid chromatography and mass spectrometry
TEM: Transmission electron microscopy
UV–Vis: UV–Visible
XRD: X‐ray diffraction

## 1 Introduction

Across routine care and clinical studies, therapy combinations that include proteasome inhibitors, immunomodulatory agents, and monoclonal antibodies targeting CD38 or SLAMF7 have delivered high response rates in both newly diagnosed and relapsed multiple myeloma [1]. However, most patients eventually relapse because of intratumoral heterogeneity, clonal evolution under therapeutic pressure, and acquired drug resistance [2]. Therefore, there is a vital need to develop novel therapeutic agents with distinct mechanisms of action to overcome drug resistance and attain better durable disease control in multiple myeloma.

Natural medicinal plants serve as valuable reservoirs of potential antitumor agents and display promising efficacy in cancer therapy. In our prior drug screening studies, berberine (BBR) and nitidine chloride (NC) were identified as promising candidates with potent anti‐multiple myeloma activity [3, 4]. BBR has been a well‐known component of traditional Chinese medicine for more than 2000 years [5], and it has been studied in recent years because of its broad clinical application and numerous therapeutic activities (e.g., anti‐inflammatory [6], anti‐cancer [7], anti‐cardiovascular disease [8], and hypo‐uric acid [9]), as well as low toxicity [10]. NC, an alkaloid with a phenanthridine backbone, can be extracted from several plants, including Zanthoxylum nitidum, also known as “Liang‐Mian‐Zhen” [11]. In recent years, NC has been reported to exhibit excellent anti‐tumor effects, indicating the potential of NC as an anti‐tumor drug [12]. However, the clinical translation of BBR and NC as therapeutic agents for anti‐multiple myeloma is hindered by inherent drawbacks, including low bioavailability, poor aqueous solubility and stability, rapid in vivo metabolism, and ambiguous pharmacokinetic pathways [13]. In order to overcome the shortcomings, several methods are utilized, including nanotechnology [14]. Recently, the use of self‐assembled nanotechnology to improve natural substances has gradually become a hot research topic [15]. The rate of drug release and half‐life can be regulated through direct self‐assembly of active monomers to fabricate carrier‐free nanodrugs [16, 17]. Building on this concept, multiple carrier‐free BBR self‐assembly/co‐assembly systems have been reported. BBR can co‐assemble with flavonoid glycosides into nanoparticles or nanofibers with tunable architectures and bioactivities, and it forms stable nanostructures with small aromatic acids, such as cinnamic acid or rhein, to enhance antibacterial/anti‐biofilm performance [18]. More recently, polyphenol‐driven co‐assemblies (BBR‐tannic acid; BBR‐Epigallocatechin gallate(EGCG)) and phytochemical co‐assemblies (BBR‐curcumin) have been utilized to enhance stability, tissue retention, and therapeutic efficacy without inert carriers [19]. Importantly, BBR‐centered self‐assembled nanodrugs have also been adapted for oncology [20]. Thus, carrier‐free self‐assembly/co‐assembly strategies may help alleviate key limitations of BBR‐based therapeutics, including poor solubility and dose‐limiting side effects, However, whether BBR can stably co‐assemble with NC, and whether such BBR/NC assemblies confer therapeutic advantages and mechanistic benefits in multiple myeloma, has not yet been systematically reported.

Ferroptosis is an iron‐dependent, non‐apoptotic form of cell death characterized by mitochondrial shrinkage and lipid peroxidation accumulation [21]. Recent studies have highlighted that targeting ferroptosis‐associated proteins or employing ferroptosis inducers can effectively overcome drug resistance in various tumor types [22]. In gastric cancer, FSP1 acts as a ferroptosis suppressor that enables chemoresistance, and pharmacologic FSP1 inhibition (iFSP1) has been shown to synergize with chemotherapy in resistant models [23]. In KRAS‐mutant tumors, rewiring polyamine metabolism can potentiate KRAS inhibitor‐induced ferroptosis and improve responses across resistant cells, organoids, and xenografts [24]. In multiple myeloma, induction of ferroptosis has also been reported to resensitize tumor cells to bortezomib, suggesting ferroptosis‐based intervention as a promising strategy to counter bortezomib resistance [25]. Nuclear receptor subfamily 2 group F member 2 (NR2F2/COUP‐TFII), an orphan nuclear receptor transcription factor, has been implicated as an upstream regulator of ferroptosis [26]. Prior work has demonstrated that NR2F2 can directly bind to the GPX4 promoter in brain metastasis cells, connecting NR2F2 to GPX4‐dependent ferroptosis control [27]. Despite the role of NR2F2 in ferroptosis regulation in other cancers, its function and druggability in multiple myeloma—especially in the context of bortezomib resistance—remain unexplored.

In this study, we developed a novel carrier‐free, self‐assembled nanomedicine composed of BBR and NC, termed BBR/NC‐SAPs, to address the pressing clinical challenge of bortezomib resistance in multiple myeloma. This design improves drug solubility and leverages the intrinsic ferroptosis‐inducing potential of both agents. Through comprehensive proteomic and molecular analyses, we identified NR2F2 as a direct molecular target mediating the effect of ferroptosis. Our findings establish a new ferroptosis‐based therapeutic strategy that targets the NR2F2–GPX4 axis, providing a promising approach for overcoming drug resistance in refractory multiple myeloma.

## 2 Results

### 2.1 Physicochemical Characterization of BBR‐NC Self‐Assemblies

To characterize the self‐assembled nanoparticles formed by BBR and NC, we utilized transmission electron microscopy (TEM), dynamic light scattering (DLS), ζ‐potential measurements, time‐resolved hydrodynamic size (stability) analysis, Fourier‐transform infrared (FTIR) spectroscopy, UV–vis spectroscopy, and X‐ray diffraction (XRD). TEM showed irregular, compact nanoparticles with primary diameters of approximately 60 nm (Figure 1A). DLS revealed a monomodal hydrodynamic size distribution centered at approximately 90 nm (Figure 1B) and a surface zeta potential of approximately −12 mV (Figure 1C). The hydrodynamic diameter remained essentially unchanged over 24 h (approximately 90 nm), indicating good colloidal stability in aqueous dispersion. To further evaluate the colloidal stability of BBR/NC‐SAPs under biologically relevant conditions, we examined their hydrodynamic diameter and polydispersity index (PDI) by DLS after incubation in biological media at 37°C. Compared with the initial particle size, BBR/NC‐SAPs demonstrated no obvious increase in hydrodynamic diameter or PDI during the observation period, and no visible precipitation was observed (Figure 1D). These results indicate that BBR/NC‐SAPs maintain suitable colloidal stability under the assayed biologically relevant conditions, supporting their suitability for subsequent in vitro and in vivo experiments. FTIR spectra exhibited pronounced broadening near approximately 3400 cm^−1^ (consistent with hydrogen‐bonding), non‐additive band shapes and coupling across 1100–1300 cm^−1^ with a new broad band around approximately 1200 cm^−1^, and intensified features between 1500–1700 cm^−1^, collectively supporting π‐π interactions and/or charge‐transfer contacts in the assembled state (Figure 1E). UV–vis spectroscopy further corroborated assembly, as the sharp monomeric bands of BBR (approximately 220/270 nm) and NC (approximately 220/270/350 nm) were replaced by a smoothed, non‐additive profile, including a broad absorption >300 nm, consistent with electronic delocalization and level broadening in nanoparticles rather than a simple physical mixture (Figure 1F). XRD displayed several sharp Bragg reflections (approximately 12°, 16°, 24°, 28° 2θ), indicative of a crystalline packing motif rather than an amorphous aggregate (Figure 1G). In summary, BBR and NC form stable, crystalline nanoparticles with a well‐defined structure, mainly governed by π‐π stacking interactions.

**FIGURE 1:**
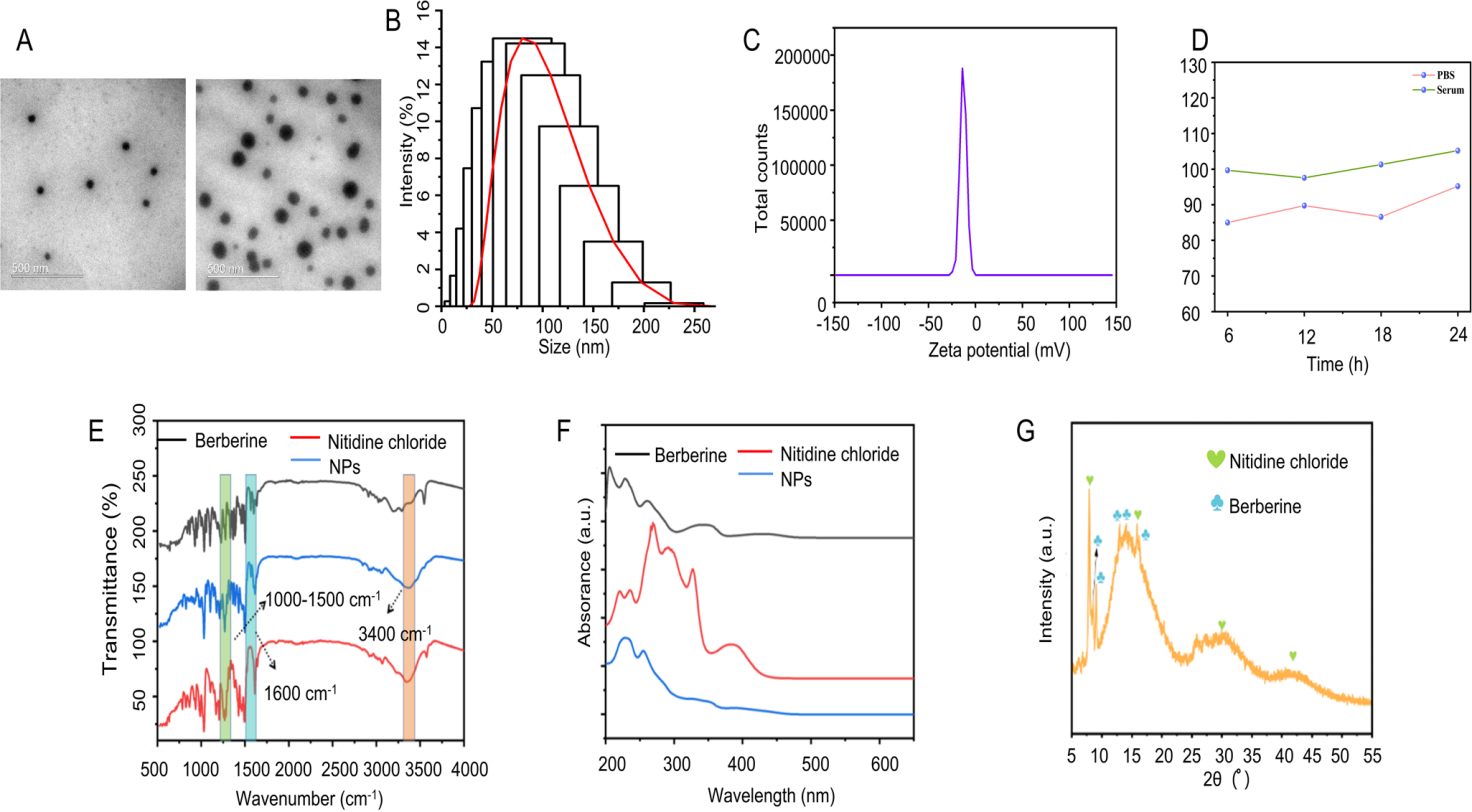
Characterization of berberine (BBR)/nitidine chloride (NC) self‐assembled nanoparticles (BBR/NC‐SAPs). (A) Transmission electron microscopy (TEM) image shows the irregular morphology of the BBR/NC‐SAPs with an average individual particle size of approximately 60 nm. (B) Dynamic light scattering (DLS) measurement shows that the hydrodynamic diameter of the BBR/NC‐SAPs is approximately 90 nm. (C) Zeta potential analysis indicates a surface charge of approximately −12 mV for the BBR/NC‐SAPs. (D) Stability assessment via DLS demonstrates that the hydrodynamic size of the BBR/NC‐SAPs remains stable at approximately 90 nm over 24 h. (E) Fourier‐transform infrared (FTIR) spectroscopy spectra. Key observations supporting successful self‐assembly include. 1) Broadening of the peak at approximately 3400 cm^−1^, suggesting hydrogen bonding between the O‐H groups of BBR and the Cl^−^ or N atoms of NC. 2) The appearance of a new broad peak around 1200 cm^−1^ and altered peak profiles in the 1100–1300 cm^−1^ range, indicating changes in the molecular bonding environment. 3) Enhanced and broadened peaks in the 1500–1700 cm^−1^ region, indicative of *π–π* stacking or charge‐transfer complex formation. (F) UV–vis absorption spectra. The spectrum of the BBR/NC‐SAPs is non‐superimposable with those of individual BBR or NC. The characteristic sharp peaks of both free drugs are replaced by a smooth, broadened profile, suggesting electronic delocalization and energy level broadening due to molecular interaction and self‐assembly. (G) X‐ray diffraction (XRD) pattern. The presence of several sharp diffraction peaks confirms the highly crystalline nature of the BBR/NC self‐assembled particles.

### 2.2 Atomistic Molecular Dynamics (MD) Predicts π‐Stacking‐Driven Co‐Assembly of BBR and NC Into Nanoclusters

To predict the self‐assembly mechanism of BBR and NC, we conducted atomistic MD simulations in a mixed aqueous/DMSO environment (Figure 2A). The simulation box (9 × 9 × 9 nm^3^) initially contained BBR and NC at a 1:1 mass ratio (31 BBR and 30 NC molecules) together with water/DMSO at a 78.8:1 molar ratio and charge‐balancing Cl^−^, followed by 100 ns production runs (298.15 K, 1 bar) utilizing PME electrostatics, a 10 Å van der Waals cutoff, and standard thermostat/barostat settings. Across the trajectory, BBR and NC rapidly aggregated from dispersed state into numerous nanoclusters and reached a quasi‐steady state by 100 ns (Figure 2B). The formation of larger, single particles was not observed within this simulation timescale, which may be attributable to electrostatic repulsion because both BBR and NC carry net positive charges (Figure 2C).

**FIGURE 2:**
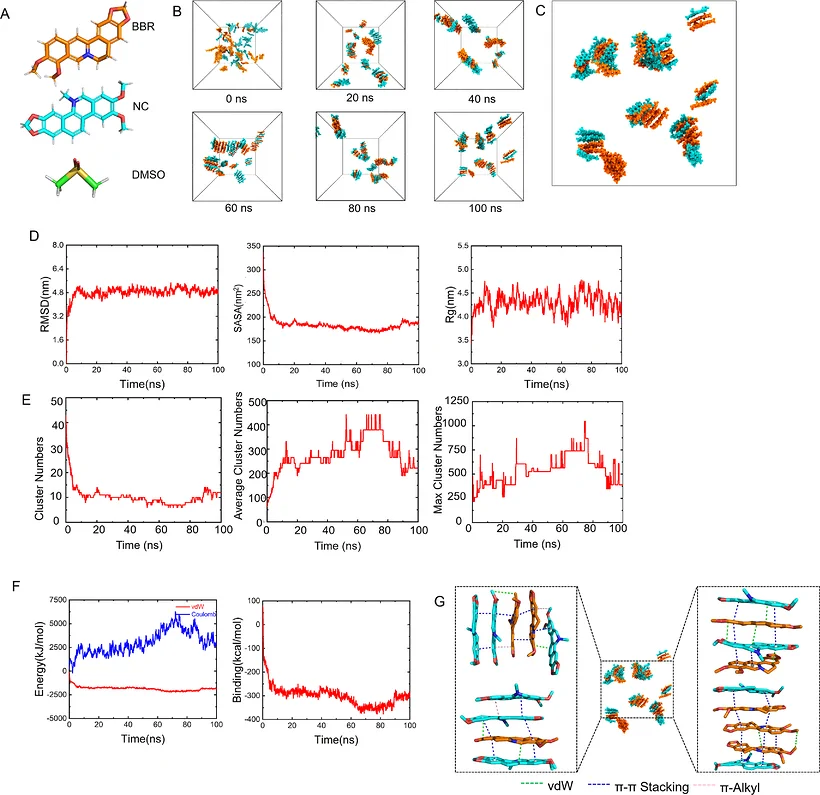
Molecular dynamics simulation reveals the self‐assembly process, stability, and driving forces of berberine (BBR)/nitidine chloride (NC) self‐assembled nanoparticles (BBR/NC‐SAPs). (A) Chemical structures of the key components: BBR (orange), NC (cyan), and the co‐solvent dimethyl sulfoxide (DMSO). (B) Representative snapshots of the self‐assembly process over 100 ns. BBR and NC molecules, shown in the VDW representation, spontaneously aggregate from a dispersed state to form numerous nanoclusters. Water, DMSO molecules, and Cl^−^ counterions are omitted for clarity. (C) Morphology of the final self‐assembled BBR/NC‐SAPs at the end of the simulation, revealing multiple compact nanoclusters rather than a single converged aggregate. (D) Temporal evolution of structural parameters during self‐assembly, including root mean square deviation (RMSD), radius of gyration (Rg), and solvent‐accessible surface area (SASA). RMSD stabilization indicates global equilibration of the system, Rg fluctuation reflects the dynamics of numerous dispersed nanoclusters, and SASA reduction indicates decreased solvent exposure during molecular aggregation. (E) Cluster analysis of the BBR/NC self‐assembly process. The time‐dependent changes in cluster number, average atom number per cluster, and atom number of the largest cluster demonstrate that BBR and NC progressively coalesce from dispersed molecules into multiple stable nanoclusters. (F) MM/PBSA‐based energy analysis of BBR/NC intermolecular association, including van der Waals, electrostatic, and overall binding free‐energy contributions. (G) Detailed view of the key intermolecular interactions stabilizing BBR/NC‐SAPs, including van der Waals forces, π‐π stacking, and π‐alkyl interactions.

The time‐dependent MD descriptors further supported the occurrence of self‐assembly. The complex root mean square deviation (RMSD) increased within the first 0–10 ns and then stabilized around approximately 4.8 nm, suggesting that the system reached a relatively equilibrated state. The radius of gyration fluctuated between 3.25–4.75 nm, consistent with the presence of several dispersed clusters rather than a single compact aggregate. In parallel, the solvent‐accessible surface area (SASA) was reduced and plateaued near approximately 186.6 nm^2^, indicating progressive burial of hydrophobic surfaces within compact assemblies (Figure 2D).

Because global RMSD alone cannot fully explain a multicomponent self‐assembling system that forms several nanoclusters, we further conducted cluster analysis to quantitatively characterize the self‐assembly process (Figure 2E). At the beginning of the simulation, BBR and NC molecules were widely dispersed, resulting in a large number of small clusters. As the simulation proceeded, the number of clusters rapidly decreased, implying progressive intermolecular association and coalescence of small aggregates. After approximately 40 ns, the cluster number fluctuated within a relatively narrow range of 8–12, suggesting that the system had formed several stable nanoclusters. Consistently, the average number of atoms per cluster increased rapidly during the early stage and then stabilized at approximately 221–379 atoms after 40 ns. The atom number of the largest cluster fluctuated between approximately 391–870 after 40 ns, signifying that the assembled clusters remained in a dynamic equilibrium with local association and dissociation events. These results provide direct evidence that BBR and NC spontaneously co‐assemble into several stable nanoclusters.

To further define the energetic basis of BBR/NC co‐assembly, we calculated the binding free energy employing the MM/PBSA method. Energy decomposition demonstrated that self‐assembly was primarily driven by favorable van der Waals interactions, whereas Coulombic contributions remained positive, consistent with electrostatic repulsion between like‐charged species. Nevertheless, the overall binding free energy was strongly favorable, supporting the spontaneous association between BBR and NC molecules (Figure 2F). Contact analyses further showed π‐π stacking and π‐alkyl contacts as the principal specific interactions stabilizing the co‐assemblies (Figure 2G). Overall, these MD simulations indicate that BBR and NC co‐assemble into several stable nanoclusters mainly through van der Waals forces and π‐π stacking interactions.

### 2.3 BBR/NC‐SAPs Are Efficiently Internalized by MM Cells

To provide direct evidence for the cellular uptake of BBR/NC‐SAPs, we conducted time‐course confocal fluorescence imaging in both parental and bortezomib‐resistant multiple myeloma cells. The 8226, 8226‐BTZR, KMS‐11, and KMS‐11‐BTZR cells were incubated with BBR/NC‐SAPs for 0, 0.5, 1, 2, and 4 h, and intracellular fluorescence signals were monitored by confocal laser scanning microscopy. At 0 h, only minimal background fluorescence was observed. After 0.5 h of incubation, weak intracellular fluorescence became detectable. The fluorescence intensity gradually increased at 1 and 2 h and became markedly stronger at 4 h in all of the tested multiple myeloma cell lines (Figure S1). These results indicate that BBR/NC‐SAPs can be efficiently taken up by multiple myeloma cells in a time‐dependent manner.

### 2.4 Superior Cytotoxicity of BBR/NC‐SAPs vs. Equimass BBR+NC Mixture in MM Cells

In order to rigorously assess the anti‐myeloma activity of BBR/NC‐SAPs and, at matched total mass, to directly compare them with a simple equimass mixture of the two free drugs (BBR + NC mix), we first performed dose‐response cell viability assays. As visually represented in the heatmap, treatment with the BBR/NC‐SAPs resulted in a more pronounced decrease in cell viability across a range of concentrations as compared to an equivalent dose of conventional free BBR + NC (IC_50_, µg/mL: 8226, 0.9948 vs. 2.806; 8226‐BTZR, 0.5157 vs. 1.819; KMS‐11, 2.072 vs. 3.264; KMS‐11‐BTZR, 0.4736 vs. 2.516; Figure 3A,B). A clonogenic assay showed that BBR/NC‐SAPs significantly inhibited the self‐renewal capacity of MM cells in a concentration‐dependent manner (Figure 3C). Quantitative analysis found a marked reduction in colony formation across all of the tested cell lines following treatment. In 8226 cells, BBR/NC‐SAPs at concentrations of 2, 4, 6, 8, and 10 µg/mL reduced colony formation to 57%, 37%, 18.5%, 5.8%, and 0.2% of the control, respectively (p < 0.01). A similar trend was observed in bortezomib‐resistant 8226‐BTZR cells, where colony formation was suppressed to 47%, 28%, 10.6%, 4%, and 0.1% at the same concentrations (p < 0.01). Likewise, in KMS‐11 cells, BBR/NC‐SAPs treatment resulted in a decrease in colony formation to 73%, 66%, 31%, 12.6%, and 3% of the control at 2, 4, 6, 8, and 10 µg/mL, respectively (p < 0.01). The bortezomib‐resistant KMS‐11‐BTZR line also exhibited pronounced sensitivity, with colony formation inhibited to 53%, 36%, 13%, 4.8%, and 0.1% under equivalent conditions (p < 0.01). A concentration‐dependent suppression of proliferation was observed in all of the tested cell lines following BBR/NC‐SAPs treatment, as quantified by an EdU assay (Figure 3D). In the 8226 and 8226‐BTZR lines, the EdU‐positive fractions were reduced from 32.6% and 49.9% in controls to 2.29% and 0.25%, respectively. Similarly, in KMS‐11 and KMS‐11‐BTZR cells, the percentage of Edu‐positive cells decreased from 32.8% and 43.9% in controls to 0.061% and 0.39%, respectively (p < 0.01 for all of the comparisons), indicating that BBR/NC‐SAPs effectively inhibit cell proliferation activity. Our investigation into the cell death mechanism revealed BBR/NC‐SAPs as a potent inducer of mitochondrial membrane potential (ΔΨm) loss, as measured by JC‐1 staining (Figure 3E). A pronounced shift from JC‐1 aggregates to monomers was observed upon treatment with up to 10 µg/mL BBR/NC‐SAPs. Notably, the JC‐1 monomer percentage increased substantially in both parental and resistant cell lines, from 7.67% to 52.3% (8226); 8.37% to 75.4% (8226‐BTZR); 8.75% to 69.4% (KMS‐11); and 8.43% to 73.4% (KMS‐11‐BTZR). Taken together, these data demonstrate that, at equal mass dosing, BBR/NC‐SAPs outperform simple co‐dosing of the free drugs in killing MM cells and independently curb proliferation and disrupt ΔΨm, supporting BBR/NC‐SAPs as a more effective formulation/delivery strategy.

**FIGURE 3:**
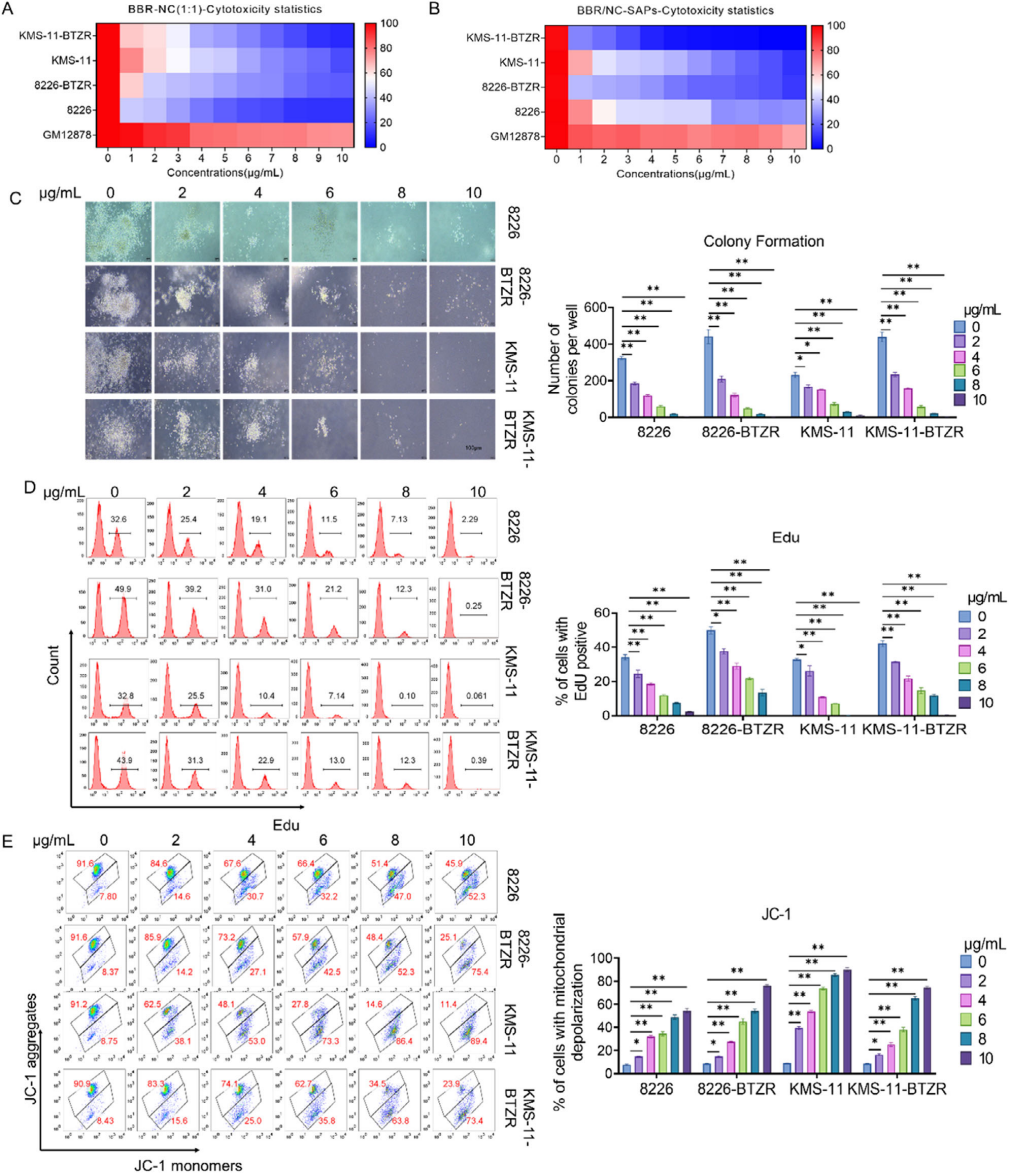
Berberine (BBR)/nitidine chloride (NC) self‐assembled nanoparticles (BBR/NC‐SAPs) display potent and selective anti‐myeloma activity by inhibiting proliferation, clonogenicity, DNA synthesis, and inducing mitochondrial dysfunction. (A) Multiple myeloma cells (sensitive: 8226, KMS‐11; bortezomib‐resistant: 8226‐BTZR, KMS‐11‐BTZR) and normal B cells (GM12878) were treated for 48 h with a concentration gradient (0, 2, 4, 6, 8, and 10 µg/mL) of free BBR and NC combination (1:1). (B) Multiple myeloma cells (sensitive: 8226, KMS‐11; bortezomib‐resistant: 8226‐BTZR, KMS‐11‐BTZR) and normal B cells (GM12878) were treated for 48 h with a concentration gradient (0, 2, 4, 6, 8, and 10 µg/mL) of BBR/NC‐SAPs. (C) Cells were treated with the indicated concentrations (0, 2, 4, 6, 8, and 10 µg/mL) of BBR/NC‐SAPs. Colony formation assay demonstrating the potent, dose‐dependent inhibition of clonogenic survival. (D) Cells were treated with the indicated concentrations (0, 2, 4, 6, 8, and 10 µg/mL) of BBR/NC‐SAPs. Analysis of DNA synthesis by EdU incorporation assay utilizing flow cytometry. The percentage of EdU‐positive (green) cells decreased in a dose‐dependent manner upon treatment. (E) Assessment of mitochondrial membrane potential by JC‐1 staining employing flow cytometry. Treatment with BBR/NC‐SAPs induced a dose‐dependent loss of mitochondrial membrane potential. Data are presented as the mean ± SD from three independent experiments. Statistical significance was determined by a one‐way ANOVA followed by Dunnett's multiple comparison test versus the control group. ^*^ *p* < 0.05, ^**^ *p* < 0.01.

### 2.5 Proteomic Profiling Reveals Ferroptosis Activation in MM Cells Treated With BBR/NC‐SAPs

In order to determine the mechanism by which BBR/NC‐SAPs induce cell death in MM cells, we performed a proteomics experiment (Figure S2A). We found that in BBR/NC‐SAPs‐treated MM cells, 592 proteins were downregulated and 450 proteins were upregulated. The top 10 upregulated and downregulated proteins are shown in Figure S2B. Notably, among the significantly downregulated proteins, we identified both GPX4 and NR2F2. The subcellular localization analysis of the differentially expressed proteins found a broad distribution across several cellular compartments. The majority of proteins were localized in the Cytoplasm (35.44%) and Membrane (25.31%), collectively accounting for over 60% of the total. A substantial proportion of proteins was also found in the Extracellular region (11.59%) and Mitochondrion (7.05%). Other notable localizations included the Endoplasmic reticulum (5.94%), Golgi apparatus (4.31%), Lysosome (2.74%), Ribosome (2.63%), Nucleolus (1.85%), Centrosome (2.07%), Nuclear membrane (0.62%), and Peroxisome (0.45%) (Figure S2C). Kyoto Encyclopedia of Genes and Genomes (KEGG) pathway enrichment analysis implicated ferroptosis as a key mechanism (Figure S2D). Similarly, Wikipathway analysis also indicated the activation of ferroptosis (Figure S2E). The results from the three categories collectively indicate a coordinated biological response. To execute differentiation and reconstruction programs, cells extensively produce structural and ribosomal proteins (MF) that operate efficiently within key compartments, particularly mitochondria (CC). 1) Biological Process (BP) (Figure S2F): The differentially expressed genes are predominantly enriched in protein synthesis, particularly mitochondrial translation, and cellular structural reorganization, including intermediate filament cytoskeleton organization. This collectively shows that the experimental treatment drives an active cellular reprogramming focused on epithelial barrier formation and tissue repair. 2) Cellular Component (CC) (Figure S2G): Cellular component analysis demonstrated that the gene products are highly localized to the ribosome, especially mitochondrial ribosomes and large ribosomal subunits. This further confirms the broad activation of protein synthesis and specifically underscores the key role of mitochondria as central organelles for biosynthesis and energy metabolism in this response. 3) Molecular Function (MF) (Figure S2H). The PPI network results highlight the mechanistic relevance of ferroptosis. The identification of a distinct, highly interactive protein module enriched for ferroptosis‐related genes indicates that ferroptosis is not only an associated process but constitutes a core regulatory component of the system's response to the experimental treatment (Figure S2I). These results indicate that ferroptosis was significantly induced in MM cells treated with BBR/NC‐SAPs.

### 2.6 BBR/NC‐SAPs Induce Ferroptosis in MM Cells in a Concentration‐ and Time‐Dependent Manner

To further evaluate the mechanisms underlying the selective cytotoxicity of the BBR/NC‐SAPs, we assessed multiple well‐established forms of regulated cell death utilizing MM cells as experimental models. Various cell death inhibitors were used, including autophagy inhibitors (3‐methyladenine [3‐MA]), an apoptosis inhibitor (Z‐VAD‐FMK), a necroptosis inhibitor (Nec‐1), a ROS inhibitor (acetylcysteine, NAC), and a ferroptosis inhibitor (Fer‐1). Given that our prior findings revealed the ability of BBR/NC‐SAPs to modulate iron homeostasis in cells, our results indicate that ferroptosis is the predominant pathway (Figure 4A). After treatment with 2–10 µg/mL of BBR/NC‐SAPs, the proportion of Fe^2+^ increased from 15.3% to 89.7% in 8226 cells, from 19.1% to 76.2% in 8226‐BTZR cells, from 15.5% to 91.2% in KMS‐11 cells, and from 33.7% to 90.6% in KMS‐11‐BTZR cells (Figure 4B). Given that lipid ROS accumulation is a key characteristic of ferroptosis, we next examined its levels via flow cytometry following treatment with BBR/NC‐SAPs. We observed that exposure to 2–10 µg/mL of BBR/NC‐SAPs induced a dramatic increase in lipid ROS levels—nearly 500‐fold in 8226 cells, 100‐fold in 8226‐BTZR cells, 600‐fold in KMS‐11 cells, and 250‐fold in KMS‐11‐BTZR cells, providing functional evidence that BBR/NC‐SAPs trigger ferroptosis (Figure 4C). We next sought to confirm this result by assessing the levels of key ferroptosis‐related proteins following treatment with various concentrations of BBR/NC‐SAPs, as well as with various BBR/NC‐SAP exposure times. Our analysis found a concentration‐ and time‐dependent upregulation of ferroptotic proteins (HO‐1, ACSL4, NRF2, and DMT1) and a concomitant downregulation of ferroptotic proteins (xCT, Keap1, GPX4, and DHODH) (Figure 4D and Figure S3). In summary, BBR/NC‐SAPs activate ferroptosis in MM cells in a concentration‐ and time‐dependent manner.

**FIGURE 4:**
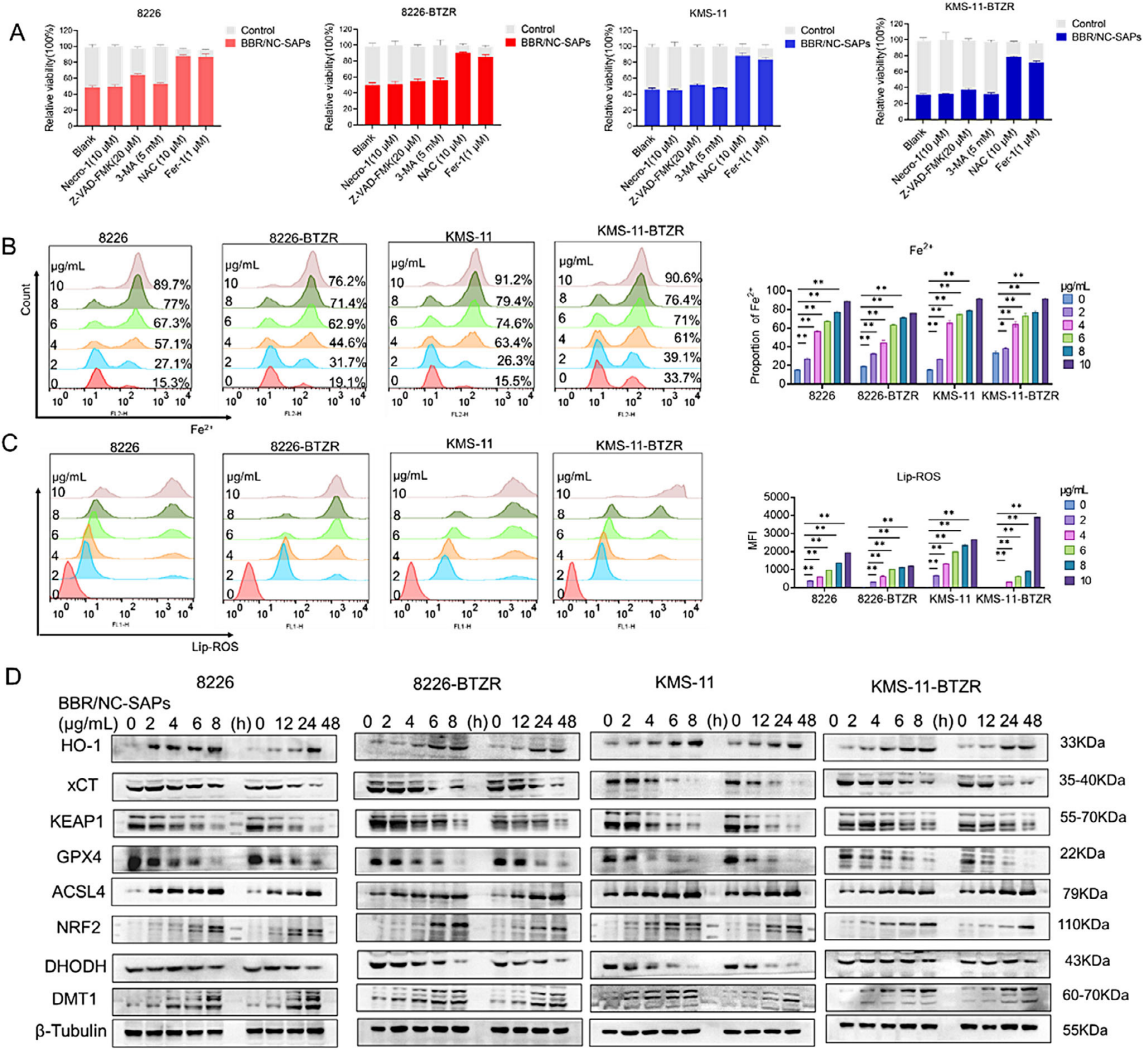
Berberine (BBR)/nitidine chloride (NC) self‐assembled nanoparticles (BBR/NC‐SAPs) induce ferroptosis in multiple myeloma (MM) cells in a concentration‐ and time‐dependent manner. (A) Numerous cell death pathway inhibitors were utilized to assess the mode of BBR/NC‐SAPs‐induced cytotoxicity in multiple myeloma cells. Inhibitors included 3‐methyladenine (3‐MA, autophagy), Z‐VAD‐FMK (apoptosis), necrostatin‐1 (Nec‐1, necroptosis), N‐acetylcysteine (NAC, ROS), and ferrostatin‐1 (Fer‐1, ferroptosis). Among these, only Fer‐1 significantly attenuated cell death, indicating a ferroptotic mechanism. (B) Intracellular Fe^2+^ levels were evaluated and demonstrated a similar concentration‐ and time‐dependent accumulation, consistent with the ferroptotic phenotype. (C) Lipid ROS levels were measured by flow cytometry and showed a concentration‐ and time‐dependent increase following BBR/NC‐SAPs treatment. (D) Western blot analysis showing dose‐ and time‐dependent upregulation of pro‐ferroptotic proteins (HO‐1, ACSL4, NRF2, and DMT1) and downregulation of anti‐ferroptotic proteins (xCT, Keap1, GPX4, and DHODH) after treatment with BBR/NC‐SAPs. Data are presented as the mean ± SD from three independent experiments. Statistical significance was determined by a one‐way ANOVA followed by Dunnett's multiple comparison test versus the control group. ^*^ *p* < 0.05, ^**^ *p* < 0.01.

### 2.7 Identification of NR2F2 as a Direct Target of BBR/NC‐SAPs

To examine how BBR/NC‐SAPs trigger ferroptosis, we analyzed the targets of BBR and NC in MM cells that were previously identified by SPR‐HPLC/MS. This analysis identified NR2F2 and BCHE as common targets (Figure 5A). Given the association between NR2F2 and the ferroptosis‐related protein GPX4, we hypothesized that BBR/NC‐SAPs activate ferroptosis through NR2F2. To test this hypothesis, we examined the interaction between BBR/NC and NR2F2 utilizing molecular docking, molecular dynamics simulations, cellular thermal shift assay (CETSA), and surface plasmon resonance (SPR). According to the molecular docking result, the ALA217 and PHE221 residues on the NR2F2 receptor form hydrophobic interactions with NC. The ARG228, GLU214, ARG218, LEU386, TYR191, PRO190, GLU225, SER404, and GLY403 residues on the NR2F2 receptor form van der Waals interactions with NC. The PHE221 residue on the NR2F2 receptor forms a Pi‐Pi stacked interaction with NC. The ALA217, ARG218, and PHE221 residues on the NR2F2 receptor form hydrophobic interactions with BBR. The LEU386, GLU214, SER405, TYR191, PRO190, SER222, GLU225, SER404, ARG228, and GLY403 residues on the NR2F2 receptor form van der Waals interactions with BBR. The PHE221 residue on the NR2F2 receptor forms a Pi‐Pi stacked interaction with BBR (Figure 5B). The molecular dynamics results showed that both the NR2F2‐BBR and NR2F2‐NC complex systems displayed stable binding with favorable hydrogen bond interactions (Figure 5C). Since the NR2F2‐NC complex exhibited lower fluctuation and greater stability throughout the simulation, we concluded that the NC molecule binds more favorably to the NR2F2 target protein. To further confirm the binding between BBR/NC and NR2F2, we conducted SPR assays. The results confirmed that both BBR and NC bind to NR2F2, with NC displayed a stronger binding signal (Figure 5D). Sensorgrams were globally fitted using a 1:1 Langmuir kinetic binding model (A + B ⇌ AB) to derive the association rate constant (k_a), dissociation rate constant (k_d), and equilibrium dissociation constant (K_D; K_D = k_d/k_a). The fitted kinetic parameters showed high‐affinity binding between NR2F2 and both small molecules, with K_D values of 3.2 × 10^−8^ M for NC‐NR2F2 and 7.3 × 10^−8^ M for BBR‐NR2F2, signifying a stronger interaction between NC and NR2F2 (Figure 5E). We also found that after binding, the NR2F2 protein degraded more rapidly with increasing temperature in the BBR‐treated group than in the NC‐treated group, indicating that NC forms a tighter and more stable interaction with NR2F2 (Figure 5F and Figure S4). Taken together, our experimental results establish that both BBR and NC engage in direct binding interactions with the NR2F2 protein in MM cells.

**FIGURE 5:**
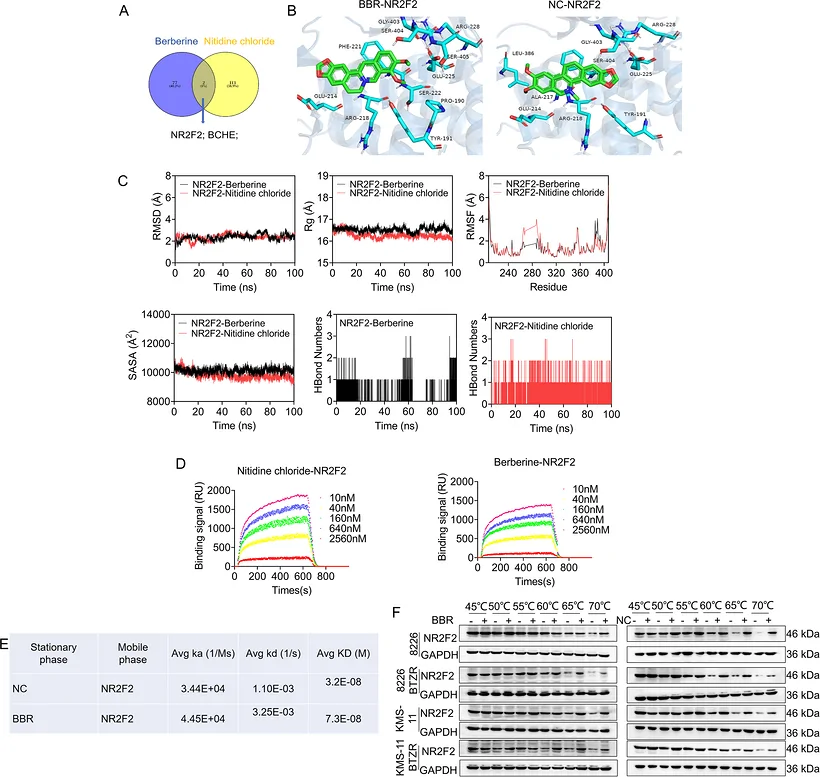
Identification and validation of NR2F2 as a direct binding target of berberine (BBR)/nitidine chloride (NC) self‐assembled nanoparticles (BBR/NC‐SAPs) in multiple myeloma (MM) cells. (A) Venn diagram or target overlap analysis demonstrating NR2F2 and BCHE as shared targets of both BBR and NC. (B) Molecular docking models illustrating the predicted binding sites of BBR and NC on the NR2F2 protein. Key interacting residues include Pro190, Tyr191, Glu214, Ala217, Arg218, Phe221, Ser222, Glu225, Arg228, Leu386, Gly403, Ser404, and Ser405. (C) Molecular dynamics (MD) simulations showing that both NR2F2‐BBR and NR2F2‐NC complexes are stable, with the NC complex showing reduced RMSF values and stronger hydrogen bonding, indicating more favorable interaction. (D) A surface plasmon resonance (SPR) assay confirming direct binding of both BBR and NC to NR2F2, with NC displaying an elevated binding affinity. (E) Representative SPR sensorgrams and fitted kinetic parameters for NC and BBR binding to NR2F2. Sensorgrams were globally fitted using a 1:1 Langmuir model. The calculated K_D values were 3.2 × 10^−8^ M for NC–NR2F2 and 7.3 × 10^−8^ M for BBR–NR2F2, indicating a stronger binding of NC to NR2F2. Data are representative of three independent experiments. (F) Cellular thermal shift assay (CETSA) revealing enhanced thermal stability of NR2F2 upon NC treatment compared to BBR, indicating stronger and more stable binding of NC to NR2F2.

### 2.8 NR2F2 as a Key Mediator in BBR/NC‐SAPs‐induced Cell Ferroptosis

In order to determine if BBR/NC‐SAPs activate ferroptosis via NR2F2, we knocked out the NR2F2 protein in MM cells using CRISPR‐Cas9 and assessed the functional outcome by evaluating cytotoxicity, colony formation, mitochondrial membrane potential, and key ferroptosis markers, including ferroptosis‐related proteins, lipid ROS, and Fe^2+^ levels. We employed two distinct CRISPR‐Cas9 sgRNAs to knock out NR2F2. The results showed that, as compared to the control group, NR2F2 loss phenocopied the effects of the BBR/NC‐SAPs and occluded further response (Figure 6A), and NR2F2 knockout significantly impaired the clonogenic capacity of 8226 cells, decreasing it from approximately 300 to 80 colonies per well. Notably, this reduction in colony formation did not decrease further upon treatment with BBR/NC‐SAPs. A similar trend was observed in the 8226‐BTZR, KMS‐11, and KMS‐11‐BTZR cell lines (Figure 6B and Figure S5A). The results from the Edu assay showed that knockout of NR2F2 markedly decreased the percentage of Edu‐positive cells across all of the tested cell lines—from 30% to 10% in 8226 cells, 42% to 16% in 8226‐BTZR cells, 28% to 6% in KMS‐11 cells, and 35% to 10% in KMS‐11‐BTZR cells. Notably, this reduction in proliferation was not further decreased upon the addition of BBR/NC‐SAPs (Figure 6C and Figure S5B). The JC‐1 assay demonstrated that NR2F2 knockout markedly increased the percentage of JC‐1 monomers (consistent with mitochondrial depolarization) in all of the tested cell models. Specifically, the monomer rates increased from 9.8% to 32.6% (8226), 5.49% to 54.2% (8226‐BTZR), 7.7% to 20.1% (KMS‐11), and 13.6% to 45.9% (KMS‐11‐BTZR). This effect eventually plateaued, as subsequent treatment with BBR/NC‐SAPs did not induce a further increase (Figure 6D and Figure S5C), indicating that NR2F2 is essential for the function of BBR/NC‐SAPs. Moreover, further investigation into the ferroptosis pathway revealed that in NR2F2‐knockout cells the Fe^2+^ levels were significantly increased in all of the cell lines—from 13.2% to 51.9% in 8226 cells, 18.3% to 62.8% in 8226‐BTZR cells, 23.3% to 57.6% in KMS‐11 cells, and 16.5% to 59.7% in KMS‐11‐BTZR cells. This increase eventually plateaued and was not further enhanced by the addition of BBR/NC‐SAPs (Figure 6E and Figure S5D). NR2F2 knockout also induced a dramatic upsurge in lipid ROS levels, nearly 400‐fold in 8226 cells, 500‐fold in 8226‐BTZR cells, 700‐fold in KMS‐11 cells, and 450‐fold in KMS‐11‐BTZR cells. This induction was also not augmented by subsequent treatment with BBR/NC‐SAPs (Figure 6F and Figure S5E). The expression of ferroptosis‐associated proteins was not further altered (Figure 6G,H and Figure S5F). Collectively, these loss‐of‐function findings demonstrate that NR2F2 is indispensable for BBR/NC‐SAPs‐induced ferroptosis. To definitively solidify this target engagement through a gain‐of‐function approach, we subsequently performed genetic rescue experiments.

**FIGURE 6:**
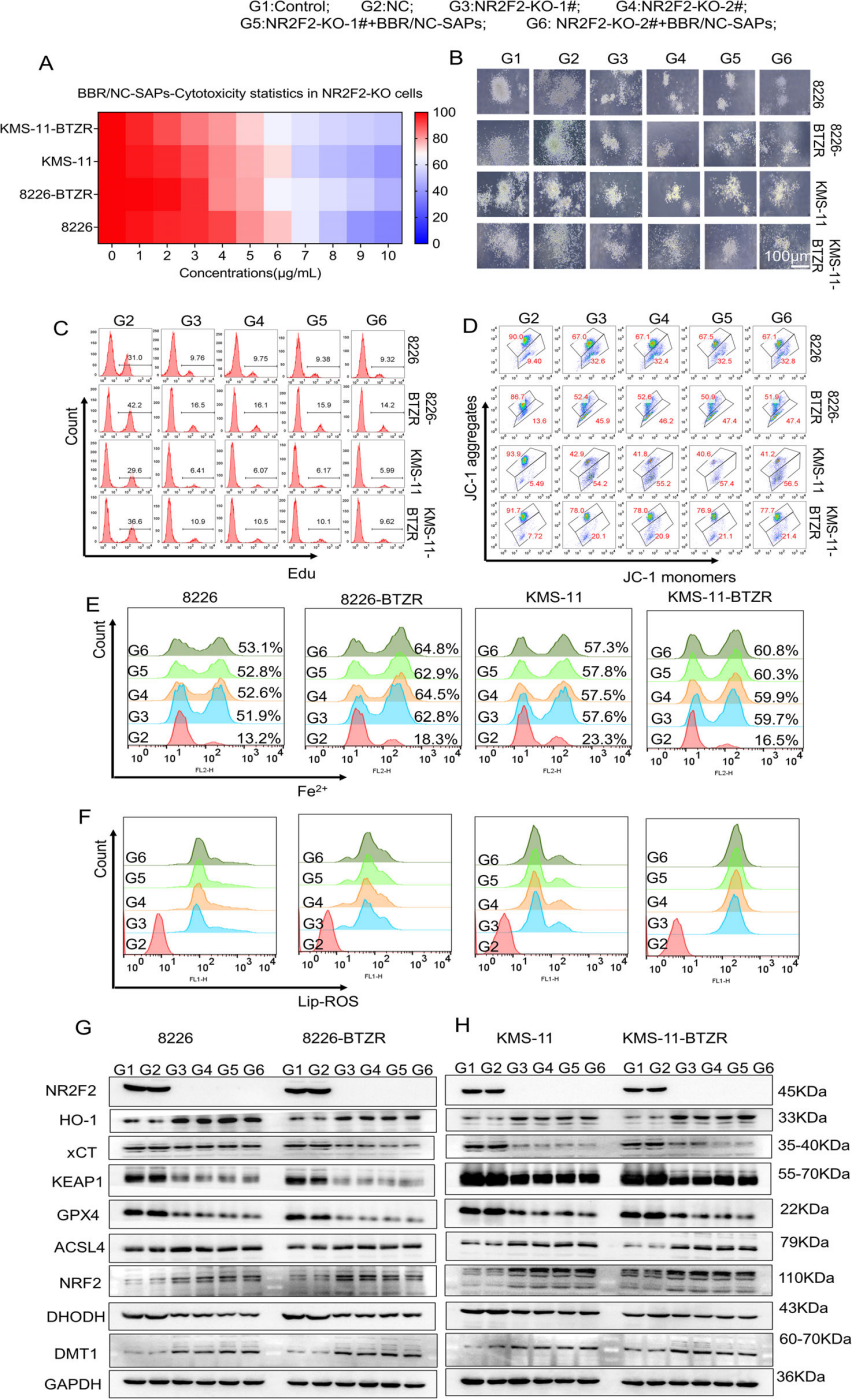
NR2F2 is essential for berberine (BBR)/nitidine chloride (NC) self‐assembled nanoparticles (BBR/NC‐SAPs)‐induced ferroptosis in multiple myeloma (MM) cells. (A) Cytotoxicity assays showing that NR2F2‐knockout cells locked the survival threshold and abolished the dosage‐dependent cytotoxicity of BBR/NC‐SAPs as compared to the control. (B) Colony formation assays revealing that NR2F2 knockout preserves clonogenic potential after BBR/NC‐SAPs treatment. (C) Analysis of DNA synthesis by EdU incorporation assay utilizing flow cytometry, demonstrating that NR2F2 knockout preserves cell proliferation potential after BBR/NC‐SAPs treatment. (D) Assessment of mitochondrial membrane potential (ΔΨm) via JC‐1 or similar dye, denoting that NR2F2 deletion prevents membrane depolarization triggered by BBR/NC‐SAPs. (E) Quantification of intracellular Fe^2+^ showing blocked iron accumulation in NR2F2‐deficient cells upon BBR/NC‐SAPs treatment. (F) Lipid ROS levels measured by flow cytometry demonstrating that BBR/NC‐SAPs fail to elevate lipid peroxidation in NR2F2‐knockout cells. Ferroptosis‐related protein levels measured by western blot showing that BBR/NC‐SAPs fail to elevate lipid peroxidation in NR2F2‐knockout cells (G, 8226 and 8226‐BTZR; H, KMS‐11 and KMS‐11‐BTZR).

### 2.9 NR2F2 Reconstitution Restores the Responsiveness of NR2F2‐Deficient KMS‐11‐BTZR Cells to BBR/NC‐SAPs

To further validate if NR2F2 is functionally required for BBR/NC‐SAPs‐induced ferroptosis, we reconstituted NR2F2 expression in NR2F2‐knockout KMS‐11‐BTZR cells using a lentiviral overexpression system. Successful NR2F2 restoration was confirmed prior to functional assays (Figure S6A). Compared with NR2F2‐knockout cells, NR2F2‐reconstituted KMS‐11‐BTZR cells regained sensitivity to treatment with BBR/NC‐SAPs. Colony formation assays demonstrated that BBR/NC‐SAPs markedly inhibited clonogenic growth in a concentration‐dependent manner after NR2F2 re‐expression (Figure S6B). Consistently, EdU incorporation assays revealed a dose‐dependent reduction in the proliferative activity of NR2F2‐rescued cells following exposure to BBR/NC‐SAPs (Figure S6C). We next evaluated whether NR2F2 restoration also recovered the ferroptotic response to BBR/NC‐SAPs. Flow cytometric analysis showed that BBR/NC‐SAPs induced a concentration‐dependent accumulation of intracellular Fe^2+^ in NR2F2‐reconstituted KMS‐11‐BTZR cells (Figure S6D). Lipid ROS levels were also elevated after treatment with BBR/NC‐SAPs, indicating activation of iron‐dependent lipid peroxidation (Figure S6E). In parallel, western blot analysis revealed that BBR/NC‐SAPs remodeled ferroptosis‐related proteins toward a ferroptosis‐activated state after NR2F2 re‐expression, as reflected by the upregulation of pro‐ferroptotic markers and the suppression of anti‐ferroptotic proteins, including GPX4. These findings indicate that NR2F2 re‐expression restores the ferroptotic response of NR2F2‐deficient KMS‐11‐BTZR cells after treatment with BBR/NC‐SAPs (Figure S6F, G). These rescue results complement the NR2F2‐knockout data and show that NR2F2 re‐expression restores the ability of BBR/NC‐SAPs to suppress clonogenicity and proliferation while promoting ferroptotic features in bortezomib‐resistant MM cells. Collectively, these findings further support NR2F2 as a direct binding target and an essential functional mediator through which BBR/NC‐SAPs regulate the GPX4‐associated ferroptosis axis.

### 2.10 BBR/NC‐SAPs Effectively Inhibit MM Growth in Vivo Through Ferroptosis

To assess the therapeutic efficacy and safety of BBR/NC‐SAPs in MM treatment, we performed two in vivo studies using BALB/c nude and B‐NDG mice with 8226‐BTZR tumor xenografts. These mice were randomized into eight groups and treated with one of the following: 1) vehicle control, 2) bortezomib (2 mg/kg), 3) BBR (5 mg/kg) + NC (5 mg/kg), 4) BBR (10 mg/kg) + NC (10 mg/kg), 5) BBR/NC‐SAPs (10 mg/kg), 6) BBR/NC‐SAPs (20 mg/kg), 7) BBR/NC‐SAPs (10 mg/kg) + Fer‐1 (2 mg/kg), and 8) BBR/NC‐SAPs (20 mg/kg) + Fer‐1 (2 mg/kg) every other day for 20 days. We were able to successfully establish a bortezomib‐resistant model, as evidenced by the failure of 2 mg/kg bortezomib to inhibit tumors induced by 8226‐BTZR cells in mice. Our results showed that the lowest dose[(BBR (5 mg/kg) + NC (5 mg/kg)] was able to inhibit tumor growth (Figure 7A,B) and reduce tumor weight (Figure 7C) as compared to controls, highlighting its potent anti‐tumor effects. BBR/NC‐SAPs have a better antitumor effect than the free drugs at the same concentrations. Further, co‐administration of the specific ferroptosis inhibitor Fer‐1 with BBR/NC‐SAPs significantly suppressed their cytotoxic effect on MM mice, providing further mechanistic evidence that ferroptosis is the primary mode of cell death. No significant difference in mouse body weight was observed among the various treatment groups, indicating the absence of substantial toxicity at the administered drug concentrations (Figure 7D). We established a disseminated, bortezomib‐resistant MM model by inoculating 8226‐BTZR‐Luc cells into mice. Longitudinal in vivo bioluminescence imaging (BLI) showed diffuse, whole‐body signals in vehicle‐treated mice, confirming successful engraftment, and bortezomib (BTZ) treatment failed to decrease tumor burden, indicating that the model is BTZ‐refractory. In contrast, administration of the free‐drug combinations of BBR and NC at 5 mg/kg BBR + 5 mg/kg NC or 10 mg/kg BBR + 10 mg/kg NC markedly suppressed the proliferation of 8226‐BTZR‐Luc cells in vivo and (Figure 7E,F). Notably, at equivalent total doses, the BBR/NC‐SAPs yielded stronger inhibition of tumor growth and a greater survival benefit than the free‐drug combinations, suggesting enhanced absorption/bioavailability afforded by the nanoformulation (Figure 7G). IHC detection of Ki‐67, ACSL4, NR2F2, and GPX4 expression in tumor tissues found that ACSL4 was significantly elevated after treatment with BBR/NC‐SAPs, and this increase was reversed by Fer‐1. In contrast, NR2F2, Ki67, and GPX4 were significantly reduced after treatment with BBR/NC‐SAPs, and Fer‐1 was able to reverse these decreases (Figure S7A). To preliminarily assess the in vivo safety of BBR/NC‐SAPs, we further conducted histopathological analysis of major organs after treatment. During the treatment period, H&E staining of major organs, including the heart, liver, spleen, lung, and kidney, revealed no obvious pathological abnormalities. These results indicated that BBR/NC‐SAPs exert potent anti‐myeloma activity without causing obvious systemic toxicity or major‐organ histopathological injury under the treatment conditions tested (Figure S7B). In summary, we show that BBR/NC‐SAPs display superior anti‐tumor efficacy as compared to the free drugs in a bortezomib‐resistant MM mouse model, and this effect is mainly mediated through the induction of ferroptosis.

**FIGURE 7:**
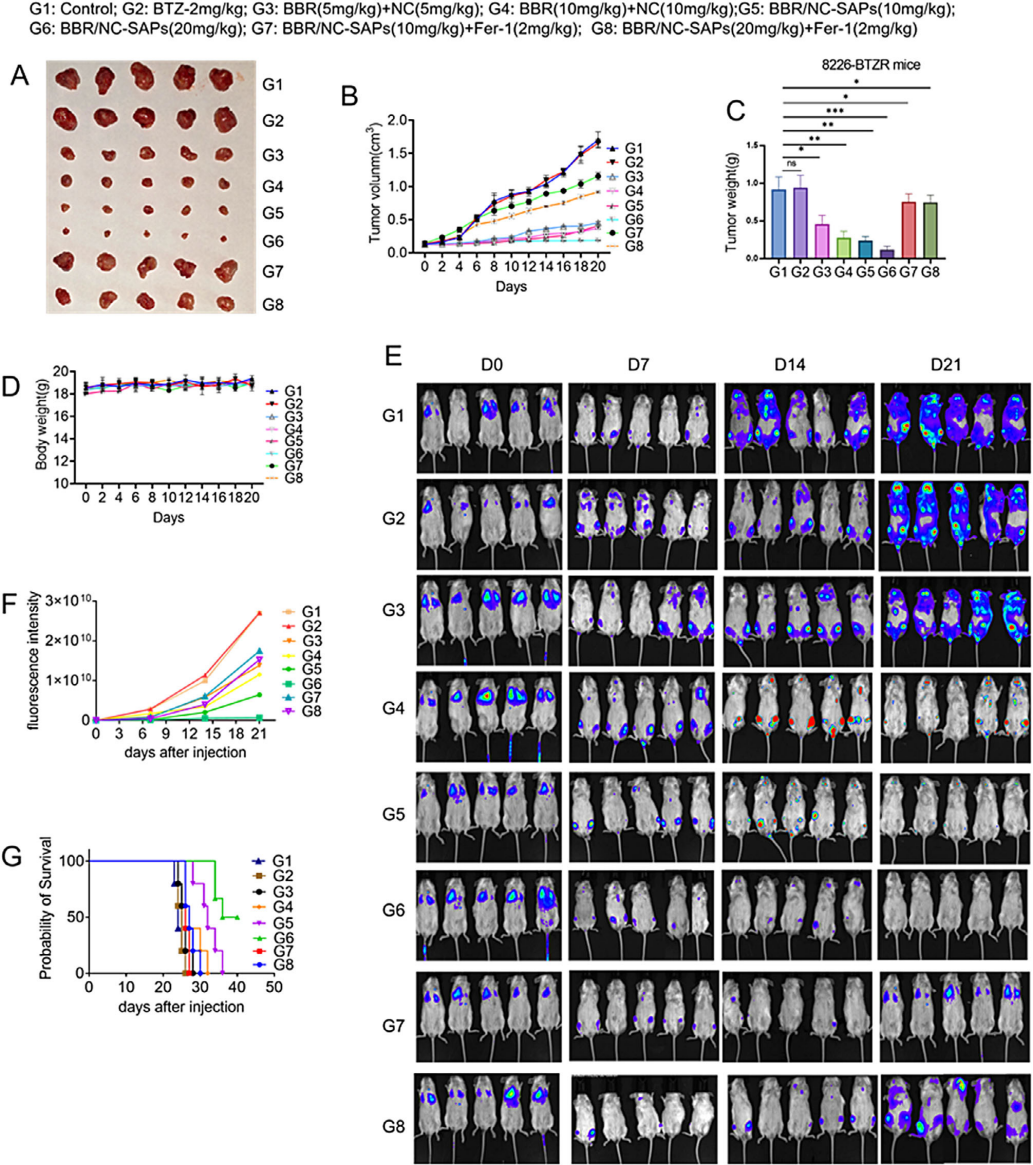
Berberine (BBR)/nitidine chloride (NC) self‐assembled nanoparticles (BBR/NC‐SAPs) effectively inhibit multiple myeloma (MM) growth in vivo through ferroptosis. (A) Representative images of tumors isolated from mice (*n* = 5). (B) Growth curves of tumor volume in mice during treatment with one of the following: bortezomib (2 mg/kg), BBR (5 mg/kg) + NC (5 mg/kg), BBR (10 mg/kg) + NC (10 mg/kg), BBR/NC‐SAPs (10 mg/kg), BBR/NC‐SAPs (20 mg/kg), BBR/NC‐SAPs (10 mg/kg) + Fer‐1 (2 mg/kg), or BBR/NC‐SAPs (20 mg/kg) + Fer‐1 (2 mg/kg). (C) Excised tumor weight on day 20. (D) Changes in body weight during treatment with one of the following: bortezomib (2 mg/kg) or BBR (5 mg/kg) + NC (5 mg/kg), BBR (10 mg/kg) + NC (10 mg/kg), BBR/NC‐SAPs (10 mg/kg), BBR/NC‐SAPs (20 mg/kg), BBR/NC‐SAPs (10 mg/kg) + Fer‐1 (2 mg/kg), or BBR/NC‐SAPs (20 mg/kg) + Fer‐1 (2 mg/kg) treatment. (E) Bioluminescence images at days 0, 7, 14, 21 post‐xenografting of orthotopic 8226‐BTZR‐Luc MM‐bearing mice treated with one of the following: bortezomib (2 mg/kg), BBR (5 mg/kg) + NC (5 mg/kg), BBR (10 mg/kg) + NC (10 mg/kg), BBR/NC‐SAPs (10 mg/kg), BBR/NC‐SAPs (20 mg/kg), BBR/NC‐SAPs (10 mg/kg) + Fer‐1 (2 mg/kg), and BBR/NC‐SAPs (20 mg/kg) + Fer‐1 (2 mg/kg). (F) Quantified luminescence levels of bioluminescence images. (G) Survival curve of mice in each group with the indicated drug treatment. Data are presented as the mean ± SD. Statistical significance was determined by a one‐way ANOVA followed by Dunnett's multiple comparison test versus the control group. ^*^ *p* < 0.05, ^**^ *p* < 0.01, ^***^ *p* < 0.001.

## 3 Discussion

MM is the second most prevalent hematological malignancy, characterized by the uncontrolled proliferation of plasma cells within the bone marrow [28]. Although significant progress has been attained in MM therapy, most patients ultimately develop drug resistance and experience relapse [29]. Therefore, the development of effective and innovative therapeutic strategies for MM, especially drug‐resistant MM, remains an urgent need. Carrier‐free, green, and multi‐targeted nanodrugs are possible solutions [30].

Traditional Chinese medicine has been developed for more than 5000 years, and it involves a large number of plants, herbs and spices that have the potential to act as anti‐MM drugs. BBR and NC are important active constituents of traditional Chinese medicine and have been utilized for the treatment of various diseases for over 2000 years [31]. However, their poor water solubility leads to extremely low oral bioavailability [32]. In our previous study, both BBR and NC exhibited significant therapeutic effects against MM. Thus, improving their absorption has become a central focus. In the context of absorption and function, BBR self‐assembly transforms a poorly absorbed, rapidly cleared alkaloid into carrier‐free supramolecular nanoparticles with higher apparent solubility/permeability, prolonged circulation, greater tumor accumulation, and sustained intracellular availability, thus collectively enhancing its effective bioavailability [33]. By eliminating inert excipients and enforcing π‐π/hydrophobic packing, these systems reach very high drug loading and reshape cellular uptake and trafficking, which, in turn, amplifies pharmacodynamics [34]. Recent examples, including BBR‐glycyrrhetinic acid (BBR‐GA) and BBR‐artesunate (BBR‐ART) nanoparticles, have been shown to outperform equimass free‐drug mixtures in vivo, highlighting the “drug‐as‐carrier” advantage while decreasing carrier‐related liabilities and remaining compatible with scalable manufacture [33]. Consistent with this paradigm, our BBR/NC‐SAPs achieve superior potency at matched mass dose and curb proliferation relative to a simple BBR + NC mixture, supporting self‐assembly as a practical route to upgrade BBR exposure and unlock multi‐modal antitumor mechanisms in MM.

A large body of evidence has demonstrated that ferroptosis is closely associated with various diseases, including cancer, immune disorders, neurodegenerative diseases, stroke, and multiple organ injuries [35, 36, 37]. Prior studies have reported that BBR can inhibit tumor progression by activating ferroptosis [38]. It has been shown that BBR suppresses colorectal cancer progression by inducing ferroptosis‐mediated energy metabolism disorders [39]. In addition, cardiac sirtuin1 deficiency was shown to exacerbate ferroptosis in doxorubicin‐induced cardiac injury via the Nrf2/Keap1 pathway [40]. Further, targeting ABCB6 with NC has been found to inhibit the PI3K/AKT signaling pathway, thereby promoting ferroptosis in MM [3]. Emerging evidence supports ferroptosis activation as an anti‐myeloma and anti‐resistance strategy [41]. In this study, quantitative proteomics analysis first indicated that BBR/NC‐SAPs act in MM mainly by activating ferroptosis. Consistently, ferroptosis inhibitors rescued BBR/NC‐SAP–induced cytotoxicity. Mechanistically, BBR/NC‐SAPs increased lipid reactive oxygen species and intracellular Fe^2+^. Ferroptosis‐related protein changes further aligned with an activated‐ferroptosis state. Taken together, both BBR and NC can trigger ferroptosis; in MM, enforcing ferroptosis inhibits proliferation and helps reverse drug resistance. Accordingly, BBR/NC‐SAPs activate ferroptosis in MM.

NR2F2 (COUP‐TFII) is an orphan nuclear receptor that acts as a context‐dependent oncogenic regulator, notably promoting angiogenic programs through the VEGFA/NRP1–VEGFR2 signaling pathway [42]. NR2F2 was shown to upregulate GPX4 and inhibit ferroptosis to drive acquired chemoresistance in lung cancer [43], while GPX4 inhibition restored platinum sensitivity. Because ferroptosis susceptibility is shaped by cellular lipid composition, and NR2F2 governs adipogenic/PPAR‐linked lipid programs, NR2F2 likely tunes ferroptosis thresholds across tumors [27]. GPX4 is the only known selenoprotein in mammals capable of reducing lipid hydroperoxides to their corresponding lipid alcohols within cell membranes to prevent ferroptotic cell death [44]. Accordingly, NR2F2 emerges as a promising drug target. In this study, we identified NR2F2 as the common target of BBR and NC, and its expression decreased following treatment with BBR/NC‐SAPs. NR2F2 knockout attenuated the tumor‐killing effect of BBR/NC‐SAPs, and, notably, BBR/NC‐SAPs failed to further activate ferroptosis in NR2F2‐deficient cells. Together, these findings indicate that NR2F2 is a previously unrecognized therapeutic target in MM and that BBR/NC‐SAPs activate ferroptosis via NR2F2.

In conclusion, our study presents a rationally designed, carrier‐free nanoparticle system based on traditional medicinal alkaloids that effectively induces ferroptosis and overcomes bortezomib resistance in MM. By targeting the NR2F2‐GPX4 axis, BBR/NC‐SAPs represent a promising class of ferroptosis‐activating nanomedicines with high translational potential. Nevertheless, certain limitations remain, including the need to further investigate pharmacokinetics, long‐term biosafety, and the impact on the bone marrow microenvironment. Future studies will focus on optimizing formulation strategies, expanding therapeutic applications, and evaluating clinical feasibility in more complex models.

## 4 Materials and Methods

### 4.1 Cell Lines and Materials

Multiple myeloma (MM) cells (RPMI‐ 8226 (RRID: CVCL_0014), KMS‐11 (RRID: CVCL_2989) and the 8226‐BTZR cell line were established and stored in our laboratory. KMS‐11‐BTZR cells were purchased from the MeisenCTCC（Jin hua, Zhejiang, China). GM12878 cells were purchased from the Cellverse company (iCell‐h406, Shanghai, China) and cultured in RPMI‐1640 medium supplemented with 10% fetal bovine serum. Nitidine chloride (HY‐N0498, NC) and berberine (HY‐N0716, BBR) were obtained from MedChemExpress (MCE, USA). Dimethyl sulfoxide (DMSO; Macklin, China) was used as the solvent. Ultrapure deionized water was utilized throughout the study. The following instruments were used: ultrasonic bath (KO110, Kunshan Ultrasonic Instruments, China), particle size and zeta potential analyzer (Zetasizer Ultra, Malvern Panalytical, UK), transmission electron microscope (JEM‐1200EX, JEOL, Japan), FTIR spectrometer (Nicolet iN10, Thermo Fisher Scientific, USA), UV–vis spectrophotometer (UV‐2401, Shimadzu, Japan), and X‐ray diffractometer (D8 DISCOVER, Bruker, Germany).

### 4.2 Preparation of Self‐Assembled Nanoparticles (SAPs)

BBR (5 mg) and NC (5 mg) were dissolved separately in DMSO (1 mL each) with brief sonication to obtain stock solutions. The two stocks were then combined and added dropwise into 20 mL of deionized water under ice‐bath sonication. Sonication was continued for 30 min. Dispersions were centrifuged at 10,000 rpm for 30 min, and the supernatant containing the SAPs was collected, lyophilized, and stored at 4°C until use.

### 4.3 Transmission Electron Microscopy (TEM)

SAP dispersions were diluted in water, deposited onto carbon‐coated copper grids, allowed to adsorb for 1–2 min, wicked to dryness, and imaged on a JEM‐1200EX TEM. Particle morphology was evaluated qualitatively from representative micrographs.

### 4.4 Dynamic Light Scattering (DLS) and Zeta Potential

Hydrodynamic diameter (Z‐average) and polydispersity index (PDI) were measured on a Zetasizer Ultra using disposable cuvettes. Zeta potential was determined by electrophoretic light scattering utilizing folded capillary cells. Measurements were conducted at an ambient temperature on freshly prepared dispersions. To further assess colloidal stability under biologically relevant conditions, freshly prepared BBR/NC‐SAPs were dispersed in the indicated biological media and incubated at 37°C. At predetermined time points, aliquots were collected, and the hydrodynamic diameter and PDI were measured by DLS to monitor particle‐size stability and aggregation tendency. All of the measurements were conducted in triplicate.

### 4.5 Colloidal Stability Assay

To assess short‐term colloidal stability, SAP hydrodynamic size was recorded over 24 h under ambient storage. No additional additives were introduced during the monitoring period.

### 4.6 Fourier‐Transform Infrared (FTIR) Spectroscopy

Dried samples were assessed on a Nicolet iN10 FTIR spectrometer. Spectra of individual components and corresponding SAPs were collected to evaluate spectral band shifts/broadening indicative of supramolecular interactions (e.g., hydrogen bonding and π‐π stacking).

### 4.7 UV–Vis Spectroscopy

UV–vis spectra were recorded on a Shimadzu UV‐2401 spectrophotometer for single‐component solutions and SAP dispersions. Spectral profiles were compared to identify non‐additive features characteristic of self‐assembly.

### 4.8 Powder X‐Ray Diffraction (XRD)

Dried powders of SAPs were evaluated on a Bruker D8 DISCOVER diffractometer. Diffraction patterns were utilized to distinguish amorphous versus crystalline features associated with the assembled states.

### 4.9 Cellular Uptake Assay

The cellular uptake of BBR/NC‐SAPs was analyzed by time‐course confocal laser scanning microscopy. In brief, 8226, 8226‐BTZR, KMS‐11, and KMS‐11‐BTZR cells were seeded in confocal dishes and incubated with BBR/NC‐SAPs at the indicated concentrations of 0, 0.5, 1, 2, and 4 h. At each time point, cells were collected or washed gently with PBS to remove extracellular nanoparticles, followed by fixation with 4% paraformaldehyde for 15 min at room temperature. Cell nuclei were counterstained with DAPI. Intracellular fluorescence signals derived from BBR/NC‐SAPs were then observed utilizing a confocal laser scanning microscope. Images were acquired under identical imaging parameters for all of the time points within each cell line to allow comparison of fluorescence intensity.

### 4.10 CCK‐8 Cell Viability Assay

Cell viability was evaluated utilizing a Cell Counting Kit‐8 (CCK‐8, Biosharp, Hefei, China). In brief, GM12878, 8226, 8226‐BTZR, KMS‐11, and KMS‐11‐BTZR cells were seeded into 96‐well plates at a density of 5 × 10^3^ cells per well and allowed to incubate overnight under standard culture conditions (37°C, 5% CO_2_). The cells were then treated with various formulations, including free BBR, NC, or BBR/NC‐SAPs at the indicated concentrations, and incubated for 48 h. Following treatment, 10 µL of the CCK‐8 reagent was added to each well, and incubation was continued for an additional 2 h at 37°C. Absorbance was subsequently measured at 450 nm employing a microplate reader (Biotek). Cell viability was calculated as (A_treated—A blank / A_control—A blank) × 100%, where A_treated and A_control represent the absorbance of treated and untreated control cells, respectively.

### 4.11 Colony Formation Assay

The 8226, 8226‐BTZR, KMS‐11 and KMS‐11‐BTZR cells were seeded into 6‐well plates at a density of 500 cells per well and cultured under standard conditions (37°C, 5% CO_2_) for 10–14 days. During incubation, cells were treated with the indicated concentrations of BBR/NC‐SAPs or control formulations, and the medium was refreshed every 3 days. When visible colonies (> 50 cells) had formed, the culture medium was removed, and the cells were gently washed twice with phosphate‐buffered saline (PBS), the plates were photographed, and the number of colonies was manually counted.

### 4.12 EdU Proliferation Assay

Cell proliferation was assessed utilizing a 5‐ethynyl‐2’‐deoxyuridine (EdU) incorporation assay (BeyoClick EdU Cell Proliferation Kit, Beyotime, Shanghai, China). MM cells (8226, 8226‐BTZR, KMS‐11 and KMS‐11‐BTZR) were seeded into 24‐well plates at a density of 1 × 10^5^ cells per well and treated with the indicated concentrations of BBR/NC‐SAPs or control formulations for 48 h. Subsequently, cells were incubated with a working solution of 10 µM EdU for 2 h at 37°C, fixed with 4% paraformaldehyde for 15 min, and permeabilized using 0.5% Triton X‐100 for 10 min. After washing with PBS, the cells were stained with the Apollo reaction cocktail for 30 min in the dark. Fluorescence images were captured employing a flow cytometry.

### 4.13 Mitochondrial Membrane Potential Assay

The mitochondrial membrane potential (ΔΨm) was assessed utilizing the fluorescent probe JC‐1 (MCE, USA) according to the manufacturer's protocol. MM cells (8226, 8226‐BTZR, KMS‐11, and KMS‐11‐BTZR) were seeded into 6‐well plates at a density of 2 × 10^5^ cells per well and treated with the indicated concentrations of BBR/NC‐SAPs or control formulations for 48 h. After treatment, cells were collected, washed twice with cold PBS, and incubated with 5 µg/mL JC‐1 working solution at 37°C for 30 min in the dark. Subsequently, cells were washed twice with JC‐1 buffer to remove excess dye and resuspended in 500 µL PBS for immediate flow cytometric analysis (BD FACS Calibur). The red (aggregated JC‐1, Ex/Em = 525/590 nm) and green (monomeric JC‐1, Ex/Em = 490/530 nm) fluorescence intensities were recorded. The percentage of green fluorescence was calculated to evaluate ΔΨm changes, where a decreased ratio indicates mitochondrial depolarization.

### 4.14 CRISPR‐Cas9‐Mediated Knockout of NR2F2

To generate NR2F2‐deficient MM cells, the CRISPR‐Cas9 gene‐editing system was employed. Two single‐guide RNAs (sgRNAs) targeting distinct exons of the human NR2F2 gene were designed utilizing the CRISPR design tool (https://www.thermofisher.cn/crispr/) to minimize potential off‐target effects. The sgRNA oligonucleotides were cloned into the lentiCRISPR‐v2 vector (Addgene, #52961), which co‐expresses Cas9 and the puromycin resistance gene. The target DNA sequences used were as follows: Target DNA Sequence‐1#: ACATTTGCGAACTGGCCGCG; Target DNA Sequence‐2#: CTCGATACCCATGATGTTGT; Lentiviral particles were generated by co‐transfecting HEK293T cells with the lentiCRISPR‐v2‐sgRNA construct, psPAX2, and pMD2.G packaging plasmids using Lipofectamine 3000 (Thermo Fisher Scientific, USA). After 48–72 h, viral supernatants were harvested, filtered through a 0.45‐µm membrane, and used to infect MM cells (8226, 8226‐BTZR, KMS‐11, and KMS‐11‐BTZR) in the presence of 8 µg/mL polybrene. Infected cells were selected with 2 µg/mL puromycin for 5–7 days to establish stable NR2F2‐knockout (NR2F2‐KO) cell lines. The knockout efficiency was verified by western blotting analysis.

### 4.15 Lentiviral NR2F2 Reconstitution in NR2F2‐Knockout Cells

For NR2F2 rescue experiments, NR2F2‐knockout KMS‐11‐BTZR cells were transduced with lentiviral particles carrying the full‐length human NR2F2 coding sequence. A corresponding empty vector was utilized as the negative control. Lentiviral infection was conducted according to the manufacturer's instructions in the presence of polybrene. After infection, stable cells were selected with puromycin, and NR2F2 re‐expression was verified by western blotting analysis before subsequent functional assays. The established NR2F2‐reconstituted KMS‐11‐BTZR cells were then treated with the indicated concentrations of BBR/NC‐SAPs for colony formation, EdU incorporation, lipid ROS, and Fe^2+^ detection assays.

### 4.16 Intracellular Iron (Fe^2+^) Measurement

Intracellular ferrous Fe^2+^ levels were measured using the fluorescent probe FerroOrange (Dojindo, Japan) following the manufacturer's instructions. MM cells (8226, 8226‐BTZR, KMS‐11, and KMS‐11‐BTZR) were seeded into 6‐well plates at a density of 2 × 10^5^ cells per well and treated with the indicated formulations (BBR/NC‐SAPs or controls) for 24 h. After treatment, cells were collected, washed twice with phosphate‐buffered saline (PBS), and incubated with 1 µM FerroOrange in serum‐free medium at 37°C for 30 min in the dark. After incubation, the cells were washed twice with PBS and resuspended in 500 µL of PBS for immediate analysis by flow cytometry (BD FACS Calibur). Fluorescence was collected at the PE channel. The mean fluorescence intensity (MFI) was used to quantify intracellular Fe^2+^ levels.

### 4.17 Lipid ROS Detection Assay

Lipid reactive oxygen species (lip‐ROS) levels were measured utilizing the fluorescent probe BODIPY 581/591 C11 (Thermo Fisher Scientific, USA). In brief, 8226, 8226‐BTZR, KMS‐11, and KMS‐11‐BTZR cells were seeded into 6‐well plates (2 × 10^5^ cells/well) and exposed to various treatments (BBR/NC‐SAPs, ferroptosis inducer, or ferroptosis inhibitor) for 24 h. Cells were then incubated with 2 µM BODIPY 581/591 C11 for 30 min at 37°C in the dark. After staining, cells were washed twice with PBS, resuspended in 500 µL of PBS, and analyzed using flow cytometry (BD FACS Calibur).

### 4.18 Western Blot Analysis

MM cells (8226, 8226‐BTZR, KMS‐11, and KMS‐11‐BTZR) were seeded in 6‐well plates and treated with BBR/NC‐SAPs at various concentrations (0, 2, 4, 6 and 8 µg/mL) for 48 h, or with 1 µg/mL BBR/NC‐SAPs for different time intervals (0, 12, 24, and 48 h). After treatment, cells were collected, washed twice with cold PBS, and lysed in RIPA buffer (Beyotime, Shanghai, China) supplemented with 1 mM PMSF and a protease and phosphatase inhibitor cocktail. Lysates were incubated on ice for 30 min and centrifuged at 12,000 × g for 15 min at 4°C. The supernatants were collected, and protein concentrations were determined utilizing a BCA Protein Assay Kit (Thermo Fisher Scientific, USA).

Equal amounts of total protein (30 µg) were separated on 10% SDS‐PAGE gels and transferred onto PVDF membranes (Millipore, USA). Membranes were blocked with 5% non‐fat milk in TBST (20 mM Tris‐HCl, 150 mM NaCl, 0.1% Tween‐20) for 1 h at room temperature and incubated overnight at 4°C with primary antibodies against NR2F2 (K007782P, Solarbio, Beijing, China, diluted 1:1000); HO‐1, ACSL4, NRF2, DMT1, xCT, Keap1, GPX4, and DHODH (all from Proteintech, PK30003, diluted 1:1000). After washing, membranes were incubated with HRP‐conjugated goat anti‐rabbit or anti‐mouse secondary antibodies (1:5000, Beyotime) for 1 h at room temperature. Protein bands were visualized using an enhanced chemiluminescence (ECL) detection kit (Bio‐Rad, USA) and imaged with a ChemiDoc MP system (Bio‐Rad). The relative expression levels of target proteins were quantified using ImageJ software and normalized to β‐tubulin, which served as the loading control. All of the experiments were independently repeated at least three times.

### 4.19 Target Identification

To explore the potential molecular targets of candidate compounds, a 8226‐BZTR cell lysate‐based ligand fishing assay was conducted utilizing surface plasmon resonance coupled with high‐performance liquid chromatography and mass spectrometry (SPR–HPLC–MS). In brief, the tested compound was covalently immobilized onto a sensor chip using a BioDot TM‐1520 array printer (BioDot Inc., USA). Protein lysates from bortezomib‐resistant MM (8226‐BZTR) cells were prepared in lysis buffer and adjusted to a final concentration of 200 µg/mL. The lysate was continuously flowed over the compound‐immobilized surface at 25°C, and sensorgrams were recorded in real time to monitor binding responses. Non‐immobilized sites served as background references. Proteins that specifically interacted with the compound were subsequently eluted, separated by liquid chromatography, and identified using tandem mass spectrometry (LC‐MS/MS). Candidate targets were ranked based on peptide‐spectrum matches and binding scores, and those with the highest scores were selected for validation.

### 4.20 Molecular Docking

Docking of BBR and NC to the NR2F2 ligand‐binding domain (LBD) was performed with AutoDock Vina. The receptor was prepared from the NR2F2 LBD crystal structure by removing crystallographic waters/hetero‐ligands, adding polar hydrogens, assigning Gasteiger charges, and converting to PDBQT via AutoDockTools. Ligands (BBR and NC) were built from 2D structures, enumerated at pH 7.4 (± 0.5) to select dominant protomers/tautomers, minimized with MMFF94, and converted to PDBQT with rotatable bonds automatically detected. The search space (grid box) was centered on the LBD cavity and sized to fully enclose the pocket and adjacent sub‐pockets. Docking used default scoring with an exhaustiveness of 32 and up to 20 poses per ligand; the receptor was kept rigid and ligands flexible. Poses were clustered at 2.0 Å RMSD, and the top‐ranked pose (best Vina score) consistent with key polar/hydrophobic contacts was selected for analysis. Protein–ligand interactions (H‐bonds, π‐π/π‐cation, salt bridges) were inspected in PyMOL and PLIP to confirm chemically plausible binding.

### 4.21 Molecular Dynamic Simulations

All of the atom MD simulations were conducted in GROMACS 2022 on NR2F2‐BBR and NR2F2‐NC complexes. The protein was parameterized with AMBER14SB; ligands used GAFF2 parameters generated via Antechamber with RESP‐fitted partial charges. Each complex was solvated in a cubic TIP3P water box with a 1.0 nm buffer; Na^+^/Cl^−^ ions were added to neutralize and reach 0.15 M. Long‐range electrostatics employed PME with a 1.0 nm real‐space cutoff and bonds were constrained using LINCS. After staged energy minimization (steepest‐descent 3,000 steps followed by conjugate‐gradient 2,000 steps), production runs were conducted for 100 ns in the NPT ensemble at 310 K (Nosé–Hoover thermostat) and 1 bar (Parrinello–Rahman barostat) with a 2 fs time step. Trajectories were analyzed using standard GROMACS tools to obtain RMSD, RMSF, radius of gyration (Rg), solvent‐accessible surface area (SASA), and protein–ligand hydrogen bonds.

### 4.22 Surface Plasmon Resonance (SPR) Analysis

Direct molecular interactions between the compound and target protein were confirmed by SPR utilizing a Biacore T200 system (Cytiva, Sweden). Recombinant human NR2F2 protein was immobilized on a CM5 sensor chip through amine coupling. A series of compound concentrations (0.05–5 µM) were injected over the chip surface at a flow rate of 30 µL/min in HBS‐EP buffer (10 mM HEPES, 150 mM NaCl, 3 mM EDTA, 0.005% Tween‐20, pH 7.4). Association and dissociation phases were monitored at 25°C. The equilibrium dissociation constant (K_D) was calculated by globally fitting the sensorgrams to a 1:1 Langmuir binding model using Biacore Evaluation Software. All of the measurements were conducted in triplicate to ensure reproducibility.

### 4.23 Cellular Thermal Shift Assay (CETSA)

Target engagement in cells was evaluated by CETSA. Cells (8226, 8226‐BTZR, KMS‐11, and KMS‐11‐BTZR) were treated with BBR/NC or vehicle (0.1–0.2% DMSO) for 1 h at 37°C, washed with PBS, resuspended at 1–2 × 10^6^ cells/mL in PBS with protease inhibitors, and aliquoted to PCR tubes. Aliquots were heated for 3 min across a temperature gradient (45°C–70°C, 5°C increments), then cooled to RT (3 min) and placed on ice. Cells were lysed by three freeze‐thaw cycles (liquid N2/25°C) in PBS with 0.4% NP‐40, 10 mM MgCl_2_, and DNase/RNase as needed. Soluble fractions were cleared (20,000 ×g, 20 min, 4°C) and analyzed by western blot for the target.

### 4.24 In Vivo Xenograft and Bioluminescence Imaging

Female NOD‐Prkdc^scid^ IL2rg^tm1/Bcgen^ (B‐NDG; 6–8 weeks old) mice (Biocytogen, Beijng, China) were maintained under specific‐pathogen‐free conditions with ad libitum access to food and water. All of the procedures were approved by the Institutional Animal Care and Use Committee of The Affiliated Guangdong Second Provincial General Hospital of Jinan University (Approval #: 2025‐DW‐KZ‐066‐01). For systemic engraftment, 5 × 10^6^ 8226‐BTZR cells stably expressing firefly luciferase (8226‐BTZR‐Luc) were injected intravenously. Treatments, namely, bortezomib (2 mg/kg), free BBR (5 and 10 mg/kg), NC (5 mg/kg and 10 mg/kg), and BBR/NC‐SAPs (10 and 20 mg/kg), were administered by intraperitoneal injection at the indicated schedules. Tumor burden was monitored by in vivo bioluminescence imaging after intraperitoneal injection of D‐luciferin (150 mg/kg; PerkinElmer) using an IVIS Lumina III system (PerkinElmer), and signals were quantified with Living Image software. Mice were monitored daily. Hind limb paralysis was prespecified as the humane endpoint.

Female BALB/c nude mice (4 weeks old; Guangdong Scenery Bio‐Tech Co., Ltd., Guangzhou, China) were utilized to establish subcutaneous MM xenografts. All of the animal procedures were reviewed and approved by the Institutional Animal Care and Use Committee of The Affiliated Guangdong Second Provincial General Hospital of Jinan University (Approval #: 2025‐DW‐KZ‐066‐01). The 8226 bortezomib‐resistant cells (8226‐BTZR; 1 × 10^7^ cells in sterile PBS) were injected subcutaneously into the right flank of each mouse (n = 5 per group). When tumors became palpable, tumor‐bearing mice were randomly assigned to treatment groups and received intraperitoneal injections of free BBR, NC, or BBR/NC‐SAPs every two days at the indicated doses. Body weight and tumor size were recorded every two days using Vernier calipers, and tumor volume (mm^3^) was calculated as 0.5 × length × width^2^. At the study endpoint, mice were euthanized by cervical dislocation, and the tumors were excised and processed for downstream analyses.

### 4.25 Statistical Analysis

All of the statistical analyses were conducted utilizing GraphPad Prism software. Quantitative data are presented as the mean ± standard deviation (SD) unless otherwise indicated. For in vitro experiments, data were obtained from at least three independent biological replicates. For animal experiments, the sample size is indicated in the corresponding figure legends. No data transformation was performed unless otherwise stated. When applicable, data were normalized to the corresponding control group before statistical analysis.

For comparisons between two groups, an unpaired two‐tailed Student's t test was used. For comparisons among multiple groups in which each treatment group was compared with the control group, a one‐way analysis of variance (ANOVA) followed by a Dunnett's multiple comparison test was employed. No all‐pairwise comparisons among treatment groups were performed unless otherwise stated. The significance symbols are defined as follows: ^*^ *p* < 0.05, ^**^ *p* < 0.01, and ^***^ *p* < 0.001; ns, not significant.

## Author Contributions

**Yiwen Lv**: conceptualization, Methodology, Validation, Formal analysis, Project administration; **Shuang Liu**: Methodology, Validation, Investigation, Writing – review & editing. **Hong Wu**: Conceptualization, Methodology, Validation; **Guiluan Li**: Resources, Data curation. **Ying Rong**: Conceptualization, Writing – original draft. **Guangchao Li**: software; **Yangmin Zhu**: Conceptualization, Writing – review & editing, Funding acquisition. **Qi Zhong**: Methodology, Validation, Investigation. **Ruiming Ou**: Visualization, Supervision. **Huijuan Shen**: Project administration. **Zhi Liu**: Resources, Data curation. **Jing Huang**: Conceptualization, Resources, Writing – review & editing, Funding acquisition. **Zhenwei Wang**: Conceptualization. **Pei Lin**: Conceptualization, Resources, Writing – review & editing. **Qing Zhang**: Methodology, Writing – review & editing, Funding acquisition. **Guopan Yu**: Conceptualization, Visualization, Supervision, Writing – review & editing, Funding acquisition. **Zhao Yin**: Conceptualization, Methodology, Validation, Formal analysis, Resources, Data curation, Funding acquisition.

## Funding

Guangzhou Science and Technology Plan Project (Nos. 2025A03J4290, 2025A03J4292, and 2025A03J4451); Foundation of Guangdong Second Provincial General Hospital (Nos,. YN2023‐001, TJGC‐2023005, TJGC‐2025009, TJGC‐2026004, 2024E006, 2024C001, YY2024‐004, YY2025‐007, and YY2024–012); Guangdong Basic and Applied Basic Research Foundation (2026A1515011519); Traditional Chinese Medicine of Guangdong Province (Nos. 20241009 and 20252001); Guangdong Medical Science and Technology Research (B2024119).

## Declaration of Generative AI in Scientific Writing

AI‐assisted tools were used only for language polishing, grammar checking, and improving the clarity of the manuscript text.

## Consent

The authors have nothing to report.

## Conflicts of Interest

The authors declare no conflicts of interest.

## Supporting information




**Supporting File**: advs77174‐sup‐0001‐SuppMat.docx.

## Data Availability

The data that support the findings of this study are available from the corresponding author upon reasonable request.
