# Supplementary material for: Carrier‐Free Berberine/Nitidine Chloride Self‐Assembled Nanoparticles Induce Ferroptosis to Overcome Bortezomib Resistance in Multiple Myeloma
Source: Adv Sci (Weinh). 2026 Aug 18;13(62):e77174. doi: 10.1002/advs.77174 (PMC13483133; doi:10.1002/advs.77174)
Supplement: Supplementary file 1 [file ADVS-13-e77174-s001.docx]

**Supplementary Figure 1. Time-dependent cellular uptake of BBR/NC-SAPs in multiple myeloma cells.**

Representative confocal fluorescence images of 8226, 8226-BTZR, KMS-11, and KMS-11-BTZR cells incubated with BBR/NC-SAPs for 0, 0.5, 1, 2, and 4 h. Intracellular fluorescence signals gradually increased over time in all tested cell lines, indicating time-dependent cellular accumulation of BBR/NC-SAPs. Nuclei were stained with DAPI. Scale bar, 10 μm. Images are representative of three independent experiments.

**Supplementary Figure 2**

**Quantitative proteomics demonstrates that BBR/NC SAPs exert anti-multiple myeloma effects through a ferroptosis-related mechanism.**

**(A)** Experimental workflow for quantitative proteomics analysis of multiple myeloma cells treated with BBR/NC-SAPs.

**(B)** Heatmap of the top 20 most significantly upregulated and downregulated proteins. Notably, key proteins such as GPX4 and NR2F2 were among the most significantly downregulated.

**(C)** Subcellular localization analysis of the differentially expressed proteins. The majority of proteins were localized in the Cytoplasm (35.44%) and Membrane (25.31%), with significant proportions also found in the Extracellular region (11.59%) and Mitochondrion (7.05%).

**(D)** KEGG pathway enrichment analysis. Ferroptosis was identified as one of the most significantly enriched pathways.

**(E)** Wikipathway analysis confirming the significant enrichment and activation of the ferroptosis pathway.

Shown are the top enriched terms from Biological Process (BP, **F**), Cellular Component (CC, **G**), and Molecular Function (MF, **H**). Bubble size indicates the number of enriched genes; color scale represents the -log₁₀ (FDR-adjusted P-value), with darker colors denoting higher significance.

**(I)** Protein-protein interaction (PPI) network analysis using STRING, demonstrating significant enrichment and clustering of the differentially expressed proteins within the ferroptosis pathway.

**Supplementary Figure 3**

The relative amount of HO-1, xCT, KEAP1, GPX4, ACLS4, NRF2, DHODH and DMT1 was semi-quantified by grayscale analysis after different concentrations and times of BBR/NC SAPs treatment.

**Supplementary Figure 4**

CETSA heatmap of NR2F2 in MM cells were determined in the absence and presence of BBR/NC. Normalized band intensity ratios from three independent experiments.

**Supplementary Figure 5**

Cell proliferation was assessed using EdU assay and colony formation assay. Mitochondrial function was evaluated by JC-1 staining to measure membrane potential depolarization. Key hallmarks of ferroptosis were quantified by measuring intracellular Fe²⁺ levels (using Ferro Orange) and lipid ROS accumulation (using C11-BODIPY 581/591). A. Colony formation; B. Edu; C. JC-1; D. Fe^2+^ ; E. Lip-ROS. F. The relative amount of HO-1, xCT, KEAP1, GPX4, ACLS4, NRF2, DHODH and DMT1 was semi-quantified by grayscale analysis after BBR/NC SAPs treatment in NR2F2-KO cell lines. Data in quantitative panels are presented as mean ± SD from three independent biological experiments. Statistical significance was determined by one-way analysis of variance (ANOVA) followed by Tukey’s post-hoc multiple-comparison test. *P < 0.05, **P < 0.01; ns, not significant.

**Supplementary Figure 6. NR2F2 re-expression restores BBR/NC-SAPs-induced ferroptosis in NR2F2-knockout KMS-11-BTZR cells.**

(A) Western blot analysis confirming NR2F2 re-expression in NR2F2-knockout KMS-11-BTZR cells following lentiviral NR2F2 overexpression.

(B) Representative images and quantitative analysis of colony formation in NR2F2-reconstituted KMS-11-BTZR cells treated with the indicated concentrations of BBR/NC-SAPs. NR2F2 re-expression restored the concentration-dependent inhibitory effect of BBR/NC-SAPs on clonogenic growth.

(C) Representative flow-cytometry plots and quantitative analysis of EdU incorporation in NR2F2-reconstituted KMS-11-BTZR cells after BBR/NC-SAPs treatment. BBR/NC-SAPs dose-dependently suppressed cell proliferation after NR2F2 re-expression.

(D) Flow-cytometric analysis and quantification of intracellular Fe²⁺ levels in NR2F2-reconstituted KMS-11-BTZR cells treated with increasing concentrations of BBR/NC-SAPs.

(E) Flow-cytometric analysis and quantification of lipid ROS accumulation in NR2F2-reconstituted KMS-11-BTZR cells after BBR/NC-SAPs treatment.

(F) Western blot analysis of ferroptosis-related proteins in NR2F2-reconstituted KMS-11-BTZR cells following treatment with the indicated concentrations of BBR/NC-SAPs. NR2F2 re-expression restored the ability of BBR/NC-SAPs to remodel ferroptosis-associated proteins toward a ferroptosis-activated state, including suppression of GPX4 and induction of pro-ferroptotic markers.

(G) The relative amount of HO-1, xCT, KEAP1, GPX4, ACLS4, NRF2, DHODH and DMT1 was semi-quantified by grayscale analysis after different concentrations and times of BBR/NC SAPs treatment.

Data in quantitative panels are presented as mean ± SD from three independent biological experiments. Statistical significance was determined by one-way ANOVA followed by Dunnett’s multiple-comparison test versus the control group. Western blot images are representative of three independent experiments. *P < 0.05, **P < 0.01, ns, not significant.

**Supplementary Figure 7. Histological safety evaluation and ferroptosis-related IHC analysis after BBR/NC-SAPs treatment *in vivo*.**

(A) Representative IHC staining images of Ki-67, ACSL4, NR2F2, and GPX4 expression in tumor tissues after different treatments. Scale bar: 50 μm.

(B) Representative H&E staining images of major organs, including the heart, liver, spleen, lung, and kidney, showing no obvious histopathological injury after treatment. Scale bar: 100 μm.
